# Insights from qualitative and bifurcation analysis of COVID-19 vaccination model in Bangladesh

**DOI:** 10.1371/journal.pone.0312780

**Published:** 2024-11-01

**Authors:** Md Abdul Kuddus, M. Mohiuddin, Anip Kumar Paul, Azizur Rahman

**Affiliations:** 1 Department of Mathematics, University of Rajshahi, Rajshahi, Bangladesh; 2 Department of Mathematics, Comilla University, Cumilla, Bangladesh; 3 School of Computing, Mathematics and Engineering, Charles Sturt University, Wagga Wagga, New South Wales, Australia; Stellenbosch University, SOUTH AFRICA

## Abstract

The unprecedented global impact of the 2019 coronavirus disease (COVID-19) has necessitated a comprehensive understanding of its transmission dynamics and control measures. In this study, we present a detailed analysis of a COVID-19 vaccination model tailored to the context of Bangladesh, incorporating dual-dose vaccination strategies. By employing qualitative and bifurcation analysis techniques, we investigate the equilibrium points, effective reproduction number (R0), and critical thresholds that influence the prevalence and control of COVID-19 in the region. Our findings reveal insights into the effectiveness of vaccination programs and provide a framework for developing targeted control plans. Through a rigorous examination of model parameters and sensitivity analysis, we identify key factors driving COVID-19 transmission dynamics, emphasizing the significance of vaccination rates and other critical parameters. The validation of our model against real-world data underscores its utility in informing evidence-based decision-making for managing the COVID-19 pandemic in Bangladesh and beyond.

## Introduction

Certainly, many people suffer from several infectious diseases, including tuberculosis, measles, influenza, HIV, severe acute respiratory syndrome (SARs) and malaria, and a large number of those infected patients die around the world every year. Infectious diseases are also transmissible or communicable diseases caused by various pathogens, bacteria, fungi, and viruses [1]. Even though the communicable disease-related mortality rate is gradually decreasing worldwide, it is still relatively high in poor and developing countries due to a lack of awareness, poor prevention, and inappropriate infection-controlling systems [2]. However, in the last decade, infectious diseases, such as plague, AIDS, severe acute respiratory syndrome (SARS) virus, and bird flu, emerged, and the world had to face huge challenges in controlling these diseases [3]. From the end of 2019 until now, the world has again faced a significant challenge again due to the new coronavirus (COVID-19). The coronavirus (COVID-19) first emerged in Wuhan city of Hubei province of China at the end of December 2019. Subsequently, it was identified in most of the countries in the world, with more than 770 million persons infected as of 24 September 2023 [4, 5]. It is a highly contagious disease that spreads from person to person through coughing or sneezing [6]. A COVID-19-infected person may suffer symptoms like fever, dry cough, flu, tiredness, diarrhea, sore throat, body aches, and shortness of breath [7]. The appearance of these symptoms in a person infected with COVID-19 usually takes two to fourteen days, and this long-term exposure or latent period makes this disease exceptional compared to other infectious diseases [8]. Also, people with strong immunity are mildly affected by this disease and do not need advanced treatment. But people suffering from other serious diseases, including asthma, heart disease, and diabetes, are particularly vulnerable to severe outcomes from COVID-19. These individuals are at higher risk of experiencing complications that can lead to severe illness or even death if they contract the virus [9].

To prevent the spread of the COVID-19 outbreak, the government has taken some essential steps such as local or area-based lockdown, the shutdown of all government and private organizations, diagnosis of the suspected cases, home quarantine and COVID-19 tests for suspected persons, isolation or hospitalization of infected persons, the establishment of some dedicated corona hospitals, increasing testing capacities, improving social awareness, enforcing social distancing, and launching vaccination [10].

Vaccination is a more effective way to prevent or control the spread of COVID-19. Although the COVID-19 vaccines produced by different medical agencies are now available in the world market, it was initially the most challenging task to invent a vaccine. Launching the first vaccine on the market took over one and a half years. At first, some vaccines were named Pfizer, AstraZeneca AZD1222, Moderna, and J&J Ad26.COV2.S got approval from the European and Italian medicine agencies on 13th March 2021 to be injected into the patient’s body [11]. Vaccination substantially prevents death from the virus infection and increases immunity in the human body against the virus. The vaccine efficacy was reported at about 94% for Moderna, 95% for Pfizer, 81.3% for AZD122, and 85% for J&J Ad26.COV2.S after vaccination [12–15]. The Strategic Advisory Group of Experts on Immunization (SAGE) suggests two doses of each vaccine for a complete series, with the interval between the vaccine doses being 21 days and 28 days for the Pfizer and Moderna vaccines, respectively; however, an extended time interval up to 42 days is also allowed for some cases [16].

On March 11, 2020, the World Health Organization (WHO), a global public health organization, declared COVID-19 as a global pandemic [17]. Based on Woldometer data up to July 20, 2021, 191.7 million people were infected in 213 countries around the world, with more than 4,113,054 deaths and 174.5 million recoveries [18]. The highest number of infected people, about 35 million, were reported in the USA [19]. The infection rate was increasing rapidly in some European countries, including Italy, France, England, Spain, Russia, and Germany, as well as in Asian countries, such as India [18].

In contrast, the flow of infections was shallow at the beginning of the epidemic. However, later, both infection and death rates increased dramatically in Bangladesh. The first case was detected on 8th March 2020 by the Institute of Epidemiology, Disease Control and Research (IEDCR) [20]. As per the Woldometer record, up to 13th April 2024, a total of 2.05 million infected cases have been officially identified, with 29,493 deaths and more than 2.02 million recovered [21].

To control and prevent the spread of COVID-19, Bangladesh implemented several key initiatives, including lockdowns and movement restrictions, mass vaccination campaign, public awareness, expansion of testing facilities, quarantine and isolation measures, international collaboration and aid. Despite these key initiatives, controlling and preventing the COVID-19 pandemic is becoming more challenging for Bangladesh due to its high population, limited testing facilities, poor healthcare systems, and other limitations. However, an appropriate vaccination strategy plan is expected to be more effective in preventing the spread of COVID-19. Bangladesh began its COVID-19 vaccination campaign on January 27, 2021. The campaign initially prioritized frontline workers, including healthcare professionals, police, and military personnel, as well as elderly citizens and those with pre-existing conditions. The country rolled out the vaccine nationwide after receiving its first shipments of the Oxford-AstraZeneca vaccine, produced by the Serum Institute of India [22].

Many researchers from different fields are continuously working to understand the behavior of COVID-19. In particular, numerous mathematical models have been developed to predict the spread of infectious diseases and inform strategies for controlling outbreaks in specific regions [23–30]. The Susceptible-Infected Recovery (SIR) mathematical model commonly used for various epidemics was first developed in 1927 by the famous epidemiologists Kermack and Mackendrick [31]. Zhang et al. studied COVID-19 infection on the Diamond Princess cruise ship, estimated the reproduction number of a novel coronavirus in the early stage of the outbreak, and made a prediction of daily new cases on the ship [32]. A nonlinear mathematical model was studied to understand and analyze the transmission dynamics of the COVID-19 outbreak by Riyapan et al. [33]. In order to investigate the scenarios of different levels of vaccine efficacy and explore the diseased dynamics of COVID-19, a mathematical model considering heterogeneous populations was proposed [33]. To evaluate the effectiveness of COVID-19 vaccination programs, Watson et al., 2022 estimated the number of additional lives that would have been lost if vaccines had not been distributed [34]. In [35], the authors studied a mathematical modeling approach to identify the critical factors of a vaccination program. The Partial Rank Correlation Coefficient (PRCC) technique and sensitivity analysis are conducted to pinpoint the most significant model parameters that affect transmission and disease prevalence [36]. A mathematical model is proposed to explore the potential outcomes of COVID-19 vaccination and the effects of various factors, such as vaccine type, age group eligibility, vaccination strategy, and population coverage [37]. Additionally, numerous studies have been conducted to clarify COVID-19 transmission dynamics [38–44].

Although the COVID-19 double-dose vaccination SEIR model is common in literature [45–47], we used a modified version of the SEIR model with double-dose vaccination in our study, which is a novel contribution to explore the dynamics of COVID-19 in the Bangladesh setting, as far to our knowledge. Here, we distinguished the infected population into two additional compartments: Mild (M) and Critical (C). We considered Mild (M) individuals who are infected, infectious and have mild respiratory illness symptoms such as nasal congestion, runny nose, and a sore throat. Critical (C) individuals who are infected, infectious and have severe symptoms including shortness of breath, chest discomfort and bluish face. We also incorporated first and second-dose vaccines to explore the impact of vaccination and other control strategies for reducing the burden of COVID-19 in Bangladesh. Here, we allow first- and second-dose vaccinated people to move to the latent compartment due to the loss of immunity, which was not considered in the previous modelling studies [45, 48, 49].

In the current study, we developed a modified nonlinear mathematical model (seven compartments) with double-dose vaccination in Bangladesh. Here, we performed a rigorous analysis of the system properties and solutions to predict both the early and late-time behaviour of the model. After deriving the effective reproduction number (R0) for COVID-19 using the next-generation matrix technique, we investigate the impact of relative magnitudes on the infected populations in Bangladesh. COVID-19 can spread in Bangladesh’s population only if R0>1 (epidemic) but can maintained in a population without the need for external inputs when R0 < 1. A disease-free population will result when effective reproduction number R0<1, which means the disease naturally dies out. Furthermore, if R0>1, then the COVID-19 virus persist in the Bangladesh population. Therefore, in order to control transmission, the period of infectiousness needs to be reduced until R0<1. We completed a bifurcation analysis to identify critical points where small changes in a parameter can lead to substantial shifts in the system’s behavior.

Next, we estimate the parameters to enhance the model’s predictive capabilities. We also performed a sensitivity analysis of the model outcomes and parameters and found that the contact rate of COVID-19 had the largest influence on disease prevalence and explored the impact of different interventions, including vaccination strategy, on the dynamics of the infected population in Bangladesh. In conclusion, our research findings will provide a better understanding of the changing epidemiology of COVID-19 in terms of vaccination coverage and will support future policy and planning of the disease control efforts in Bangladesh. In addition, our COVID-19 vaccination model provides insights with wide-ranging implications for global public health as the virus continues to impact populations worldwide. The model can be adapted to other settings with similar epidemiological conditions, and the analysis of the effective reproduction number (R_0_) offers a universal understanding of transmission dynamics, supporting policymakers in their efforts to control the spread.

This study is designed as follows: A brief introduction is written in Introduction section. The mathematical formulation of the compartmental model is discussed in Materials and Methods section. The effective reproduction number, the existence of equilibriums, stability analysis, parameters estimation, sensitivity analysis, and numerical simulations are performed in Results section. Finally, a brief discussion and conclusion are outlined at the end of this study.

## Materials and methods

We developed a compartmental model to examine the transmission dynamics of COVID-19 with dual dose vaccinations, where the total population size is divided into seven separated compartments such as susceptible class (S), uninfected individuals who are susceptible to COVID-19 infection. The rate of change of the susceptible population decreases as individuals move from the susceptible compartment to the Latent and first dose vaccinated compartments, First dose vaccinated class (V_1_), who received first dose vaccine and increases as susceptible individuals receive the first dose vaccine; Second dose vaccinated class (V_2_), who received second dose vaccine and increases as first dose vaccinated individuals receive the second dose vaccine; Latent class (L), those who are infected but not infectious. The latent individuals move from the susceptible compartment at a rate dependent on the transmission rate and the current number of mild and critical individuals, Mild class (M), who are infected, infectious and have mild respiratory illness symptoms such as nasal congestion, runny nose, and a sore throat. A proportion of the latent individuals develop mild symptoms and move into the Mild compartment, Critical class (C), who are infected, infectious and have severe symptoms including shortness of breath, chest discomfort, and bluish face. A proportion of the latent individuals develop critical symptoms and move into the critical compartment. The difference between Mild and Critical individuals is particularly useful in scenarios where Mild cases may be underreported or where public health interventions differ for mild versus critical cases (e.g., hospitalization for critical cases versus home isolation for mild cases). It also helps policymakers allocate resources effectively and predict the outcomes of different intervention strategies; and recovered class (R), those who are neither infectious nor susceptible, including people in treatment, isolation, no longer mixing/contacting others (e.g. no longer injecting drugs) or dead. The recovery population increases as mild, critical and second-dose vaccinated individuals recover from the disease. Assume the size of the total population at any time t is N(t), which is constant (that means the only way a person can leave the susceptible group is to be vaccinated or infected, and the only way a person can leave vaccinated or infected group is to recover due to received immunity), age, sex, race and social status do not affect the probability of being infected, and homogeneously mixed, and it can be written as:

$$N\left(t\right)=S\left(t\right)+V_{1}\left(t\right)+V_{2}\left(t\right)+L\left(t\right)+M\left(t\right)+C\left(t\right)+R\left(t\right).$$ (1)

**Fig 1:**
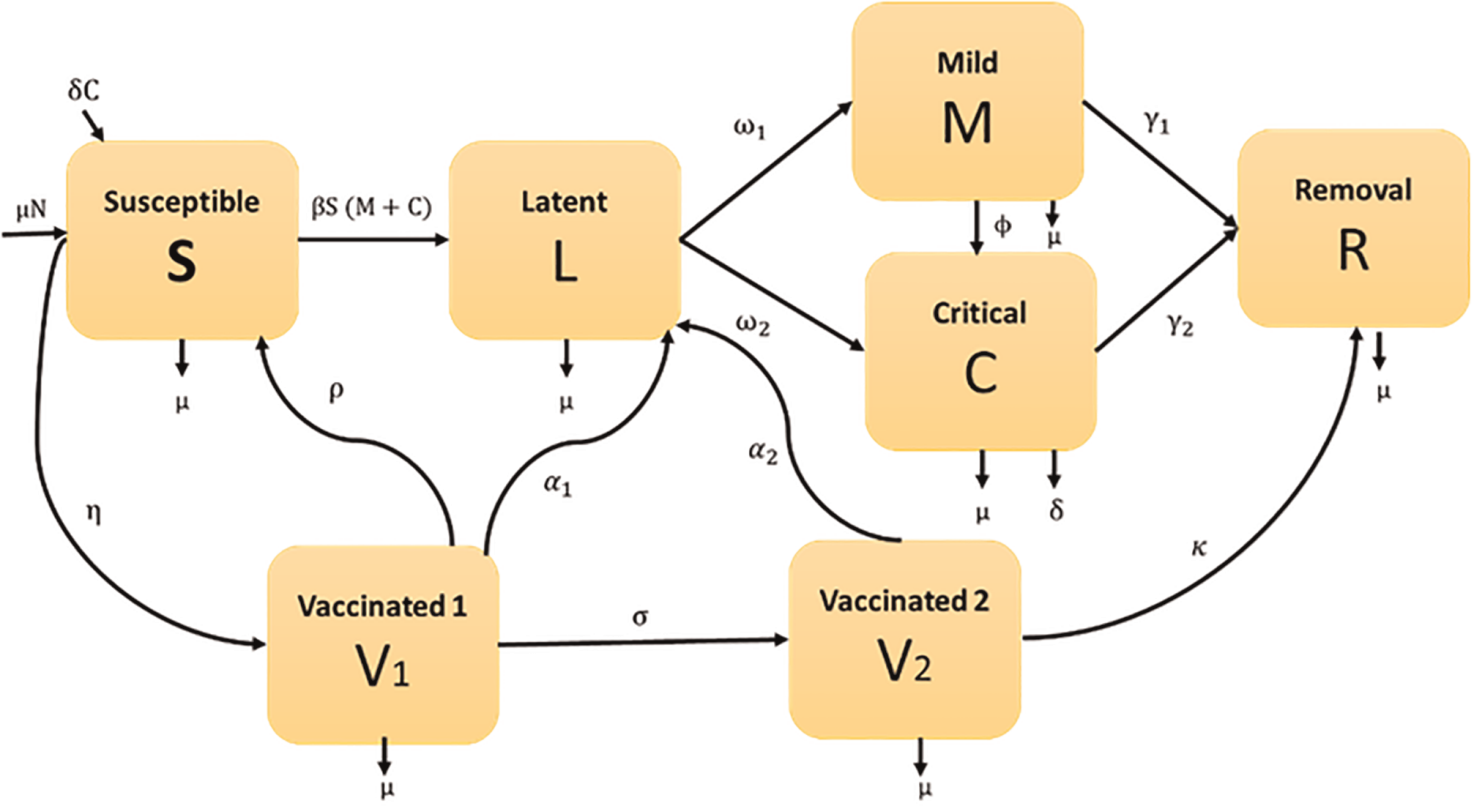
Graphic illustration of COVID-19 model in Bangladesh. English letters in boxes designate state variables (model compartments), while Greek letters represent model parameters.

Consider all deaths replaced as newborns in the susceptible compartment for keeping the population size constant. The parameter η is the rate of getting the first dose of vaccine. First-dosed vaccinated individuals V_1_ move to the susceptible group at a rate ρ due to the rate of loss of immunity and the rest of the individuals move to the second-dosed vaccinated individuals V_2_ at a rate σ. The first-dose and second-dose vaccinated persons who lose immunity are infected again and move to the Latent class with the rate of α_1_ and α_2_, respectively. The second-dosed vaccinated individual also moves to the recovery compartment at a rate *κ*. The following parameters are also used: ω_1_, ω_2_ denote the rates at which the latent population becomes infectious Mildly and Critically respectively; γ_1_, γ_2_ represent the rates at which the Mildly and Critically infected individuals are recovered due to treatment respectively; β is the contact rate between Mild or Critical class and susceptible individual; ϕ is the transfer rate of Mildly infected individuals to Critically infected individuals due to co-infection with other diseases; μ represents the birth or death rate due to natural causes which occur in all states; and δ denotes the per capita constant rate of COVID-19 related deaths. The compartmental elucidation of the model is presented in Fig 1.

From the compartmental representation of the model (Fig 1), the transmission mechanism of COVID-19 can be written by the following system of differential equations:

$$\frac{dS}{dt}=\mu N+\rho V_{1}+\delta C-\beta S\left(M+C\right)-\eta S-\mu S,$$ (2)

$$\frac{dV_{1}}{dt}=\eta S-\left(\rho +\sigma +\alpha_{1}+\mu \right)V_{1},$$ (3)

$$\frac{dV_{2}}{dt}=\sigma V_{1}-\left(\kappa +\alpha_{2}+\mu \right)V_{2},$$ (4)

$$\frac{dL}{dt}=\beta S\left(M+C\right)+\alpha_{1}V_{1}+\alpha_{2}V_{2}-\left(\omega_{1}+\omega_{2}+\mu \right)L,$$ (5)

$$\frac{dM}{dt}=\omega_{1}L-\left(\phi +\gamma_{1}+\mu \right)M,$$ (6)

$$\frac{dC}{dt}=\omega_{2}L+\phi M-\left(\gamma_{2}+\delta +\mu \right)C,$$ (7)

$$\frac{dR}{dt}=\gamma_{1}M+\gamma_{2}C+\kappa V_{2}-\mu R.$$ (8)

The above system’s (2)-(8) initial conditions are as follows:

$$S\left(0\right)\geq 0,V_{1}\left(0\right)\geq 0,V_{2}\left(0\right)\geq 0,L\left(0\right)\geq 0,M\left(0\right)\geq 0,C\left(0\right)\geq 0,R\left(0\right)\geq 0.$$ (9)

The existence and the non-negativity of the solutions of (2)-(8) and the initial conditions (9) can easily be shown for all t ≥ 0.

By summing Eqs (2)–(8), we have:

$$\frac{dN}{dt}=\frac{dS}{dt}+\frac{dV_{1}}{dt}+\frac{dV_{2}}{dt}+\frac{dL}{dt}+\frac{dM}{dt}+\frac{dC}{dt}+\frac{dR}{dt}=0.$$

Integrating this equation, we get

$$N\left(t\right)=Constant.$$

The fixed population size and positivity of solutions clearly indicate the boundedness of individually of the states S, V_1_, V_2_, L, M, C, R.

In the model (2)—(8), the recovered individuals R(t) does not present in the Eqs (2)–(7) i.e., these equations are free of R(t). Hence, to track the trajectory of disease incidence and prevalence, we can give our effort into the below-reduced system

$$\frac{dS}{dt}=\mu N+\rho V_{1}+\delta C-\beta S\left(M+C\right)-\eta S-\mu S,$$ (10)

$$\frac{dV_{1}}{dt}=\eta S-\left(\rho +\sigma +\alpha_{1}+\mu \right)V_{1},$$ (11)

$$\frac{dV_{2}}{dt}=\sigma V_{1}-\left(\kappa +\alpha_{2}+\mu \right)V_{2},$$ (12)

$$\frac{dL}{dt}=\beta S\left(M+C\right)+\alpha_{1}V_{1}+\alpha_{2}V_{2}-\left(\omega_{1}+\omega_{2}+\mu \right)L,$$ (13)

$$\frac{dM}{dt}=\omega_{1}L-\left(\phi +\gamma_{1}+\mu \right)M,$$ (14)

$$\frac{dC}{dt}=\omega_{2}L+\phi M-\left(\gamma_{2}+\delta +\mu \right)C,$$ (15)

Since the solutions of this system are non-negative and bounded the feasible solutions set for Eqs (10)–(15) enter the region:

$$D=\left\{\left(S,V_{1},V_{2},L,M,C,\right)\in R_{+}^{6}:S+V_{1}+V_{2}+L+M+C=N\right\}.$$ (16)

For the system (10)–(15), D is the positively invariant region. Consequently, we consider system (10)–(15) in the set D.

### Ethical approval

This study is based on aggregated monthly surveillance data from the COVID-19 dynamic dashboard provided by the Bangladesh Demographic and Health Survey. No confidential information was included because mathematical analyses were performed at the aggregate level. Data have no names and date of birth, so re-identification will not be possible. We believe that these data are truly de-identified and that the information listed in the line can’t be able to be attributed to individuals. We compiled data from the publicly available website https://dashboard.dghs.gov.bd/pages/covid19-bedstatus-display-graph.php. Therefore, no ethical approval is required.

## Results

### Effective reproduction number

The effective reproduction number, R_0_, is a key concept in epidemiology that represents the average number of secondary infections generated by a single infected individual in a fully susceptible population. It is a critical measure for understanding the potential spread of an infectious disease. If R_0_ > 1, the disease is likely to spread into the community and potentially cause an epidemic; if R_0_ < 1, the infection will eventually die out. R_0_ is crucial for predicting the duration and size of an epidemic and helps determine the level of intervention needed to control the spread. It also allows for the estimation of the herd immunity threshold, which is the proportion of the population that must be immune to prevent further transmission and is directly related to R_0_. In conclusion, R_0_ is a vital parameter for forecasting whether a disease will persist or be eradicated in a population.

The effective reproduction number can be derived using the next-generation matrix method (which is effectively a table describing the number of new infections generated by each individual infected with a particular strain) [50, 51]. The next-generation matrix is the product of matrices F and −V^−1^, where matrix F represents the transmission components of infected states and the matrix V describes transitions between, and out of the infected states. In this model, the infected compartments are L, M, and C. The matrices F and V for this model are given as

$$F=\left(\begin{array}{ccc} 0 & \beta S^{0} & \beta S^{0}\\ 0 & 0 & 0\\ 0 & 0 & 0 \end{array}\right)$$

and

$$V=\left(\begin{array}{ccc} -(\omega_{1}+\omega_{2}+\mu ) & 0 & 0\\ \omega_{1} & -(\phi +\gamma_{1}+\mu ) & 0\\ \omega_{2} & \phi & -(\gamma_{2}+\delta +\mu ) \end{array}\right).$$

The next-generation matrix K is given by [51]

$$\begin{array}{l} \text{K}=\text{F}(-{\text{V}}^{-1})\\ =\left(\begin{array}{ccc} 0 & {\beta \text{S}}^{0} & {\beta \text{S}}^{0}\\ 0 & 0 & 0\\ 0 & 0 & 0 \end{array}\right)\left(\begin{array}{ccc} \frac{1}{\left(\omega_{1}+\omega_{2}+\mu \right)} & 0 & 0\\ \frac{\omega_{1}}{\left(\omega_{1}+\omega_{2}+\mu \right)\left(\phi +\gamma_{1}+\mu \right)} & \frac{1}{\left(\phi +\gamma_{1}+\mu \right)} & 0\\ \frac{\omega_{1}\phi +\omega_{2}\left(\phi +\gamma_{1}+\mu \right)}{\left(\omega_{1}+\omega_{2}+\mu \right)\left(\phi +\gamma_{1}+\mu \right)\left(\gamma_{2}+\delta +\mu \right)} & \frac{\phi }{\left(\gamma_{2}+\delta +\mu \right)\left(\phi +\gamma_{1}+\mu \right)} & \frac{1}{\left(\gamma_{2}+\delta +\mu \right)} \end{array}\right)\\ =\left(\begin{array}{ccc} \frac{{\beta \text{S}}^{0}\left[\omega_{1}\left(\phi +\gamma_{2}+\delta +\mu \right)+\omega_{2}\left(\phi +\gamma_{1}+\mu \right)\right]}{\left(\omega_{1}+\omega_{2}+\mu \right)\left(\phi +\gamma_{1}+\mu \right)\left(\gamma_{2}+\delta +\mu \right)} & \frac{{\beta \text{S}}^{0}\left[\left(\phi +\gamma_{2}+\delta +\mu \right)\right]}{\left(\phi +\gamma_{1}+\mu \right)\left(\gamma_{2}+\delta +\mu \right)} & \frac{{\beta \text{S}}^{0}}{\left(\gamma_{2}+\delta +\mu \right)}\\ 0 & 0 & 0\\ 0 & 0 & 0 \end{array}\right). \end{array}$$

The spectral radius of the next generation matrix K is considered the effective reproduction number. Hence the effective reproduction number is obtained as:

$$\begin{array}{ll} {\text{R}}_{0} & \frac{{\beta \text{S}}^{0}\left[\omega_{1}\left(\phi +\gamma_{2}+\delta +\mu \right)+\omega_{2}\left(\phi +\gamma_{1}+\mu \right)\right]}{\left(\omega_{1}+\omega_{2}+\mu \right)\left(\phi +\gamma_{1}+\mu \right)\left(\gamma_{2}+\delta +\mu \right)},\\ \; & =\frac{\beta \mu \text{N}\left(\rho +\sigma +\alpha_{1}+\mu \right)\left[\omega_{1}\left(\phi +\gamma_{2}+\delta +\mu \right)+\omega_{2}\left(\phi +\gamma_{1}+\mu \right)\right]}{\left(\left(\rho +\sigma ++\mu \right)\left(\eta +\mu \right)-\eta \rho \right)\left(\omega_{1}+\omega_{2}+\mu \right)\left(\phi +\gamma_{1}+\mu \right)\left(\gamma_{2}+\delta +\mu \right)} \end{array}.$$

### Existence of equilibria

We found two equilibrium solutions: the disease-free equilibrium happens when R_0_ is less than one i.e., R_0_ < 1 and the disease endemic equilibrium happens when R_0_ is greater than one i.e., R_0_ > 1. We deliberate these in the following order.

Clearly, Eqs (10)–(15) always have an infection-free equilibrium

$$E^{0}=\left(S^{0},V_{1}^{0},V_{2}^{0},L^{0},M^{0},C^{0}\right),$$

where,

$$S^{0}=\frac{\mu N(\rho +\sigma +\alpha_{1}+\mu )}{\left(\left(\rho +\sigma +\alpha_{1}+\mu \right)\left(\eta +\mu \right)-\eta \rho \right)},$$

$$V_{1}^{0}=\frac{\mu \eta N}{\left(\left(\rho +\sigma +\alpha_{1}+\mu \right)\left(\eta +\mu \right)-\eta \rho \right)},$$

$$V_{2}^{0}=\frac{\mu \eta \sigma N}{\left(\left(\rho +\sigma +\alpha_{1}+\mu \right)\left(\eta +\mu \right)-\eta \rho \right)\left(\kappa +\alpha_{2}+\mu \right)},$$

$$L^{0}=0,$$

$$M^{0}=0,$$

$$C^{0}=0.$$

From Eqs (10)–(15) we can also determine the endemic equilibrium

$$E^{*}=\left(S^{*},V_{1}^{*},V_{2}^{*},L^{*},M^{*},C^{*}\right),$$

where

$$S^{*}=\frac{S^{0}}{R_{0}},$$

$$V_{1}^{*}=\frac{\eta S^{0}}{R_{0}(\rho +\sigma +\alpha_{1}+\mu )},$$

$$V_{2}^{*}=\frac{\sigma \eta S^{0}}{R_{0}(\rho +\sigma +\alpha_{1}+\mu )\left(\kappa +\alpha_{2}+\mu \right)},$$

$$L^{*}=\frac{\mu N\left(R_{0}-1\right)\left(\phi +\gamma_{1}+\mu \right)\left(\delta +\gamma_{2}+\mu \right)}{R_{0}\left[\left(\omega_{1}+\omega_{2}+\mu \right)\left(\phi +\gamma_{1}+\mu \right)\left(\delta +\gamma_{2}+\mu \right)-\delta \left\{\omega_{2}\left(\phi +\gamma_{1}+\mu \right)+\phi \omega_{1}\right\}\right]},$$

$$M^{*}=\frac{\mu N\omega_{1}\left(R_{0}-1\right)\left(\delta +\gamma_{2}+\mu \right)}{R_{0}\left[\left(\omega_{1}+\omega_{2}+\mu \right)\left(\phi +\gamma_{1}+\mu \right)\left(\delta +\gamma_{2}+\mu \right)-\delta \left\{\omega_{2}\left(\phi +\gamma_{1}+\mu \right)+\phi \omega_{1}\right\}\right]},$$

$$C^{*}=\frac{\mu N\left(R_{0}-1\right)\left(\omega_{2}\left(\phi +\gamma_{1}+\mu \right)+\phi \omega_{1}\right)}{R_{0}\left[\left(\omega_{1}+\omega_{2}+\mu \right)\left(\phi +\gamma_{1}+\mu \right)\left(\delta +\gamma_{2}+\mu \right)-\delta \left\{\omega_{2}\left(\phi +\gamma_{1}+\mu \right)+\phi \omega_{1}\right\}\right]}.$$ (17)

From the Eq (17), the denominator of S*, $V_{1}^{*},V_{2}^{*}$ are positive as all of the included parameter’s values are positive, whereas the denominator L*, M*, and C* depends on

$$\begin{array}{l} \left[\left(\omega_{1}+\omega_{2}+\mu \right)\left(\phi +\gamma_{1}+\mu \right)\left(\delta +\gamma_{2}+\mu \right)-\delta \left\{\omega_{2}\left(\phi +\gamma_{1}+\mu \right)+\phi \omega_{1}\right\}\right]\\ \Rightarrow \left[\left(0.034+5.78\times 10^{-4}+\frac{1}{70}\right)\left(0.3+0.02+\frac{1}{70}\right)\left(0.125+0.01+\frac{1}{70}\right)-0.125\left\{5.78\times 10^{-4}\left(0.3+0.02+\frac{1}{70}\right)+0.3\times 0.034\right\}\right]=1.139\times 10^{-3}>0 \end{array}$$ (18)

Eq (18) shows that if we put the values of ω_1_, ω_2_, γ_1_, γ_2_, μ, δ, ϕ from Table 1, we get a positive value from this equation.Eq (17) displays that the endemic equilibrium point $E^{*}=\left(S^{*},V_{1}^{*},V_{2}^{*},L^{*},M^{*},C^{*}\right)\in D$ (i.e., exist) if and only if R_0_ > 1.

**Table 1:** Explanation and estimation of COVID-19 model parameters in Bangladesh.

Parameters	Description	Values	Unit	References
**N**	Population in 2021	164,689,383	Dimensionless	[52]
**μ**	Death rate	$\frac{1}{70}$	Year^−1^	[53]
**β**	Transmission rate	2.34 × 10^−6^	Year^−1^	Fitted
**ω_1_**	Progression rate from L to M	0.013	Year^−1^	Fitted
**ω_2_**	Progression rate from L to C	0.00731	Year^−1^	Fitted
**γ_1_**	Recovered rate of mildly infected individuals	0.02	Year^−1^	[49]
**γ_2_**	Recovered rate of critically infected individuals	0.01	Year^−1^	[49]
**ϕ**	Transfer rate from mild to critical compartment	0.3	Year^−1^	[49]
**ρ**	The rate at which first dose vaccinated person moves to susceptible class	0.2	Year^−1^	[44]
**δ**	Death rate of critically infected individuals.	0.125	Year^−1^	[54]
**η**	First dose vaccination rate	0.77	Year^−1^	[55]
**α_1_**	Lose of immunity from first dose vaccinated person	0.0052	Year^−1^	Fitted
**α_2_**	Lose of immunity from second dose vaccinated person	0.0203	Year^−1^	Fitted
**σ**	Second dose vaccination rate	0.49	Year^−1^	[55]
**κ**	Recovered rate from second dose vaccinated individuals	0.80	Year^−1^	[54]

### Stability analysis

To examine the stability of the equilibria of Eqs (10)–(15), the following outcomes are proven:

### Infection-free equilibrium

***Theorem 1*:** The infection-free equilibrium of the model is locally asymptotically stable if R_0_ < 1 and unstable if R_0_ > 1.

***Proof*:** We consider the Jacobian matrix of (10)–(15) is

J=-βM+C-(η+μ)η0β(M+C)00ρ-(ρ+σ+α1+μ)σα10000-(κ+α2+μ)α200000-(ω1+ω2+μ)ω1ω2-βS00βS-(ϕ+γ1+μ)ϕ-βS+δ00βS0-(γ2+δ+μ),

which, at the infection-free equilibrium (when *R*_0_ < 1) point, E^0^, reduce to

J0=-(η+μ)η0000ρ-(ρ+σ+α1+μ)σα10000-(κ+α2+μ)α200000-(ω1+ω2+μ)ω1ω2-βS000βS0-(ϕ+γ1+μ)ϕ-βS0+δ00βS00-(γ2+δ+μ).

Hence, we can write in block matrix form as

$$J^{0}=\left(\begin{array}{cc} M_{1} & M_{2}\\ M_{3} & M_{4} \end{array}\right),$$

where,

$$M_{1}=\left(\begin{array}{ccc} -(\eta +\mu ) & \rho & 0\\ \eta & -(\rho +\sigma +\alpha_{1}+\mu ) & 0\\ 0 & \sigma & -(\kappa +\alpha_{2}+\mu ) \end{array}\right),$$

$$M_{2}=\left(\begin{array}{ccc} 0 & -\beta S^{0} & -\beta S^{0}+\delta \\ 0 & 0 & 0\\ 0 & 0 & 0 \end{array}\right),$$

$$M_{3}=\left(\begin{array}{ccc} 0 & \alpha_{1} & \alpha_{2}\\ 0 & 0 & 0\\ 0 & 0 & 0 \end{array}\right),$$

and

$$M_{4}=\left(\begin{array}{ccc} -(\omega_{1}+\omega_{2}+\mu ) & \beta S^{0} & \beta S^{0}\\ \omega_{1} & -(\phi +\gamma_{1}+\mu ) & 0\\ \omega_{2} & \phi & -(\gamma_{2}+\delta +\mu ) \end{array}\right).$$

The characteristic equation of J^0^ is

$$\begin{array}{ll} \; & \text{det}\left({\text{J}}^{0}-\lambda \text{I}\right)=\text{det}\left(\begin{array}{cc} {\text{M}}_{1}-\lambda \text{I} & {\text{M}}_{2}\\ {\text{M}}_{3} & {\text{M}}_{4}-\lambda \text{I} \end{array}\right)=0,\\ \Rightarrow & \text{det}\left({\text{M}}_{1}-\lambda \text{I}\right)\text{det}\left(({\text{M}}_{4}-\lambda \text{I}\right)-{\text{M}}_{3}{\left({\text{M}}_{1}-\lambda \text{I}\right)}^{-1}{\text{M}}_{2})=0. \end{array}$$

Since $M_{3}=\left(\begin{array}{ccc} 0 & \alpha_{1} & \alpha_{2}\\ 0 & 0 & 0\\ 0 & 0 & 0 \end{array}\right)$, then we obtain

$$\text{det}\left(M_{1}-\lambda I\right)\text{det}\left(M_{4}-\lambda I\right)=0.$$

Now we can apply the Routh-Hurwitz methods independently to the matrices M_1_ and M_4_.

Now from matrix M_1_

**Condition 1**:

$$\begin{array}{ll} \; & \text{trace}\left({\text{M}}_{1}\right)<0,\\ \; & -\left(\eta +\mu \right)-\left(\rho +\sigma +\alpha_{1}+\mu \right)-\left(\kappa +\alpha_{2}+\mu \right)<0,\\ \text{i.e.}, & \left(\eta +\mu \right)+\left(\rho +\sigma +\alpha_{1}+\mu \right)+\left(\kappa +\alpha_{2}+\mu \right)>0. \end{array}$$

**Condition 2**:

$$\begin{array}{l} \left|\begin{array}{cc} -\left(\rho +\sigma +\alpha_{1}+\mu \right) & 0\\ \sigma & -\left(\kappa +\alpha_{2}+\mu \right) \end{array}\right|+\left|\begin{array}{cc} -\left(\eta +\mu \right) & 0\\ 0 & -\left(\kappa +\alpha_{2}+\mu \right) \end{array}\right|\\ +\left|\begin{array}{cc} -\left(\eta +\mu \right) & \rho \\ \eta & -\left(\rho +\sigma +\alpha_{1}+\mu \right) \end{array}\right|>0, \end{array}$$

which gives

$$\begin{array}{ll} \; & \left(\kappa +\alpha_{2}+\mu \right)\left(\rho +\sigma +\alpha_{1}+\mu \right)+\left(\eta +\mu \right)\left(\kappa +\alpha_{2}+\mu \right)+\left(\eta +\mu \right)\left(\rho +\sigma +\alpha_{1}+\mu \right)-\rho \eta >0,\\ \text{i.e.}, & \left(\kappa +\alpha_{2}+\mu \right)\left(\rho +\sigma +\alpha_{1}+\mu \right)+\left(\eta +\mu \right)\left(\kappa +\alpha_{2}+\mu \right)+\rho \mu +\left(\eta +\mu \right)\left(\sigma +\alpha_{1}+\mu \right)>0. \end{array}$$

**Condition 3**:

$$\begin{array}{l} \text{det}\left({\text{M}}_{1}\right)<0,\\ -\left(\kappa +\alpha_{2}+\mu \right)\left(\left(\eta +\mu \right)\left(\rho +\sigma +\alpha_{1}+\mu \right)-\rho \eta \right)<0,\\ \text{i.e.},\left(\kappa +\alpha_{2}+\mu \right)\left(\rho \mu +\left(\eta +\mu \right)\left(\sigma +\alpha_{1}+\mu \right)\right)>0. \end{array}$$

From matrix M_4_

**Condition 1**:

$$\begin{array}{ll} \; & \text{trace}\left({\text{M}}_{4}\right)<0,\\ \; & -\left(\omega_{1}+\omega_{2}+\mu \right)-\left(\phi +\gamma_{1}+\mu \right)-\left(\gamma_{2}+\delta +\mu \right)<0,\\ \text{i.e.}, & \left(\omega_{1}+\omega_{2}+\mu \right)+\left(\phi +\gamma_{1}+\mu \right)+\left(\gamma_{2}+\delta +\mu \right)>0. \end{array}$$

**Condition 2**:

$$\begin{array}{l} \left|\begin{array}{cc} -\left(\phi +\gamma_{1}+\mu \right) & 0\\ \phi & -\left(\gamma_{2}+\delta +\mu \right) \end{array}\right|+\left|\begin{array}{cc} -\left(\omega_{1}+\omega_{2}+\mu \right) & {\beta \text{S}}^{0}\\ \omega_{2} & -\left(\gamma_{2}+\delta +\mu \right) \end{array}\right|\\ +\left|\begin{array}{cc} -\left(\omega_{1}+\omega_{2}+\mu \right) & {\beta \text{S}}^{0}\\ \omega_{1} & -\left(\phi +\gamma_{1}+\mu \right) \end{array}\right|>0, \end{array}$$

which gives

$$\begin{array}{l} \left(\phi +\gamma_{1}+\mu \right)\left(\gamma_{2}+\delta +\mu \right)+\left(\omega_{1}+\omega_{2}+\mu \right)\left(\gamma_{2}+\delta +\mu \right)+\left(\omega_{1}+\omega_{2}+\mu \right)\left(\phi +\gamma_{1}+\mu \right)\\ -{\beta \text{S}}^{0}\left(\omega_{1}+\omega_{2}\right)>0. \end{array}$$

**Condition 3**:

$$\begin{array}{ll} \; & \text{det}\left({\text{M}}_{4}\right)<0,\\ \; & {\beta \text{S}}^{0}\left[\omega_{1}\left(\phi +\gamma_{2}+\delta +\mu \right)+\omega_{2}\left(\phi +\gamma_{1}+\mu \right)\right]-\left(\omega_{1}+\omega_{2}+\mu \right)\left(\phi +\gamma_{1}+\mu \right)\left(\gamma_{2}+\delta +\mu \right)<0,\\ \Rightarrow & \frac{{\beta \text{S}}^{0}\left[\omega_{1}\left(\phi +\gamma_{2}+\delta +\mu \right)+\omega_{2}\left(\phi +\gamma_{1}+\mu \right)\right]}{\left(\omega_{1}+\omega_{2}+\mu \right)\left(\phi +\gamma_{1}+\mu \right)\left(\gamma_{2}+\delta +\mu \right)}<1,\\ \text{i}.\text{e}., & {\text{R}}_{0}<1. \end{array}$$

Therefore, the Routh-Hurwitz properties (It is a mathematical test that is a necessary and sufficient condition for the stability of a linear time-invariant system) are satisfied. Hence, the infection-free equilibrium E_0_ is locally asymptotically stable when R_0_ < 1.

### Endemic equilibrium

***Theorem 2*:** The endemic equilibrium of the COVID-19 model as locally asymptotically stable if R_0_ > 1.

***Proof***: We consider the Jacobian matrix of (10)–(15) is,

J=-βM*+C*-(η+μ)η0β(M*+C*)00ρ-(ρ+σ+α1+μ)σα10000-(κ+α2+μ)α200000-(ω1+ω2+μ)ω1ω2-βS*00βS*-(ϕ+γ1+μ)ϕ-βS*+δ00βS*0-(γ2+δ+μ).

The characteristic equation of the matrix J(E*) is

$$\lambda^{6}+P_{5}\lambda^{5}+P_{4}\lambda^{4}+P_{3}\lambda^{3}+P_{2}\lambda^{2}+P_{1}\lambda +P_{0}=0,$$

where,

$$A=\left(\rho +\sigma {+\alpha }_{1}+\mu \right),$$

$$B=\left(\kappa +\alpha_{2}+\mu \right),$$

$$D=\left(\omega_{1}+\omega_{2}+\mu \right),$$

$$F=\left(\phi +\gamma_{1}+\mu \right),$$

$$G=\left(\gamma_{2}+\delta +\mu \right),$$

$$H=\beta \left(M^{*}+C^{*}\right)+\left(\eta +\mu \right),$$

$$\begin{array}{l} {\text{P}}_{5}=\text{A}+\text{B}+\text{D}+\text{F}+\text{G}+\text{H},\\ =\left(\rho +\sigma +\alpha_{1}+\mu \right)+\left(\kappa +\alpha_{2}+\mu \right)+\left(\omega_{1}+\omega_{2}+\mu \right)+\left(\phi +\gamma_{1}+\mu \right)+\left(\gamma_{2}+\delta +\mu \right)+\beta \left({\text{M}}^{*}+{\text{C}}^{*}\right)\\ +\left(\eta +\mu \right)>0, \end{array}$$

$$\begin{array}{l} {\text{P}}_{4}=\left(\text{B}+\text{D}+\text{F}+\text{G}+\beta \left({\text{M}}^{*}+{\text{C}}^{*}\right)\right)\text{A}+\left(\text{D}+\text{F}+\text{G}+\text{H}\right)\text{B}+\left(\text{F}+\text{G}+\text{H}\right)\text{D}+\left(\text{G}+\text{H}\right)\text{F}+\text{GH}+\left(\eta +\mu \right)\left(\sigma +\alpha_{1}+\mu \right)\\ -\beta \text{S}\left(\omega_{1}+\omega_{2}\right), \end{array}$$

$$\begin{array}{l} {\text{P}}_{3}=\beta^{2}{\text{S}}^{*}\left({\text{M}}^{*}+{\text{C}}^{*}\right)\left(\omega_{1}+\omega_{2}\right)-{\text{R}}_{0}\text{DFG}-{\delta \omega }_{2}\beta \left({\text{M}}^{*}+{\text{C}}^{*}\right)-\text{S}\beta \left(\omega_{1}+\omega_{2}\right)\left(\text{A}+\text{B}+\text{H}\right)\\ +\left(\left(\text{D}+\text{F}+\text{G}+\beta \left({\text{M}}^{*}+{\text{C}}^{*}\right)\right)\text{A}+\left(\text{D}+\text{G}+\text{H}\right)\text{F}+\left(\text{D}+\text{H}\right)\text{G}+\text{DH}+\left(\eta +\mu \right)\left(\sigma +\alpha_{1}+\mu \right)\right)\text{B}\\ +\left(\left(\text{D}+\text{G}+\text{H}\right)\text{F}+\left(\text{D}+\text{H}\right)\text{G}+\text{DH}\right)\text{A}+\left(\left(\text{D}+\text{H}\right)\text{G}+\text{DH}-\rho \eta \right)\text{F}+\left(\text{DH}-\rho \eta \right)\text{G}-\text{D}\rho \eta , \end{array}$$

$$\begin{array}{l} {\text{P}}_{2}=\beta^{2}{\text{S}}^{*}\left({\text{M}}^{*}+{\text{C}}^{*}\right)\left(\left(\omega_{1}+\omega_{2}\right)\left(\text{A}+\text{B}\right)+\omega_{1}\left(\text{G}+\phi \right)+\omega_{2}\text{F}\right)+((-\text{AB}\left(\omega_{1}+\omega_{2}\right)\\ -\left(\text{A}+\text{B}\right)\left(\omega_{1}\left(\text{H}+\text{G}+\phi \right)+\omega_{2}\left(\text{F}+\text{H}\right)\right)+\eta \left(\omega_{1}+\omega_{2}\right)\left(\rho +\alpha_{1}\right)-\text{H}\left(\omega_{1}\left(\text{G}+\phi \right)+\omega_{2}\text{F}\right))\text{S}\\ -\delta \left({\text{M}}^{*}+{\text{C}}^{*}\right)\left(\phi \omega_{1}+\omega_{2}\left(\text{A}+\text{B}+\text{F}\right)\right))\beta +(\text{AF}\left(\text{D}+\text{G}+\text{H}\right)+\left(\text{D}+\text{H}\right)\left(\text{F}+\text{A}\right)\text{G}\\ +\text{DH}\left(\text{A}+\text{F}+\text{G}\right)-\rho \eta \left(\text{D}+\text{F}+\text{G}\right))\text{B}+\left(\left(\left(\text{D}+\text{H}\right)\text{G}+\text{DH}\right)\text{F}+\text{DGH}\right)\text{A}+\text{DFGH}\\ -\rho \eta \left(\text{GD}+\text{DF}+\text{GF}\right)-{\delta \eta \alpha }_{1}\omega_{1}, \end{array}$$

$$\begin{array}{l} {\text{P}}_{1}=\beta^{2}{\text{S}}^{*}\left({\text{M}}^{*}+{\text{C}}^{*}\right)\left(\left(\omega_{1}\left(\phi +\text{G}\right)+\omega_{2}\text{F}\right)\left(\text{A}+\text{B}\right)+\text{AB}\left(\omega_{1}+\omega_{2}\right)\right)+(((-\left(\text{H}+\text{A}\right)(\omega_{1}(\phi \\ +\text{G})+\omega_{2}\text{F})-\left(\omega_{1}+\omega_{2}\right)\left(\text{HA}-\eta \left(\rho +\alpha_{1}\right)\right))\text{B}+(\left(\left(\phi +\text{G}\right)\left(\alpha_{1}+\rho \right)+{\sigma \alpha }_{2}\right)\omega_{1}+(\left(\alpha_{1}+\rho \right)\text{F}\\ +{\sigma \alpha }_{2})\omega_{2})\eta -\left(\omega_{1}\left(\phi +\text{G}\right)+\omega_{2}\text{F}\right)\text{HA})\text{S}-\delta \left({\text{M}}^{*}+{\text{C}}^{*}\right)(\omega_{2}\left(\text{BA}+\text{BF}+\text{AF}\right)+\phi \omega_{1}(\text{A}\\ +\text{B})))\beta +(\text{DFG}\left(\text{A}+\text{H}\right)+\left(\text{FD}+\text{GD}+\text{GF}\right)\left(\beta \left({\text{M}}^{*}+{\text{C}}^{*}\right)\text{A}+\left(\eta +\mu \right)\left(\sigma +\alpha_{1}+\mu \right)\right)\\ -{\eta \delta \alpha }_{1}\omega_{2})\text{B}-\eta \left(\phi {\delta \alpha }_{1}\omega_{1}+\delta \left({\text{F}\alpha }_{1}+{\sigma \alpha }_{2}\right)\omega_{2}+\rho \text{DFG}\right)+\text{ADFGH}, \end{array}$$

$$\begin{array}{l} {\text{P}}_{0}=({\text{A}\beta }^{2}{\text{S}}^{*}\left({\text{M}}^{*}+{\text{C}}^{*}\right)\left(\omega_{1}\left(\phi +\text{G}\right)+\omega_{2}\text{F}\right)+(\left(\omega_{1}\left(\phi +\text{G}\right)+\omega_{2}\text{F}\right)\left(\rho \eta +\alpha_{1}\eta -\text{AH}\right)\text{S}-\text{A}\delta \left({\text{M}}^{*}+{\text{C}}^{*}\right)\left(\phi \omega_{1}+\omega_{2}\text{F}\right))\beta -\eta \phi {\delta \alpha }_{1}\omega_{1}\\ +\left(\text{ADGH}-\text{DG}\rho \eta -{\eta \delta \alpha }_{1}\omega_{1}\right)\text{F})\\ \text{B}+{\sigma \eta \alpha }_{2}\left(\text{S}\beta \left(\omega_{1}\left(\text{G}+\phi \right)+\omega_{2}\text{F}\right)-\delta \left(\phi \omega_{1}+\omega_{2}\text{F}\right)\right). \end{array}$$ (19)

From Eq (19), it is easy to verify that P_4_ > 0, P_3_ > 0, P_2_ > 0, P_1_ > 0 and P_0_ > 0 if M* > 0 and C* > 0. From Eq (17), it is also clear that M* and C* are positive if R_0_ > 1.

Therefore, the endemic equilibrium satisfied the Routh-Hurwitz stability properties. Hence, the endemic equilibrium point E* is locally asymptotically stable when R_0_ > 1.

**Fig 2:**
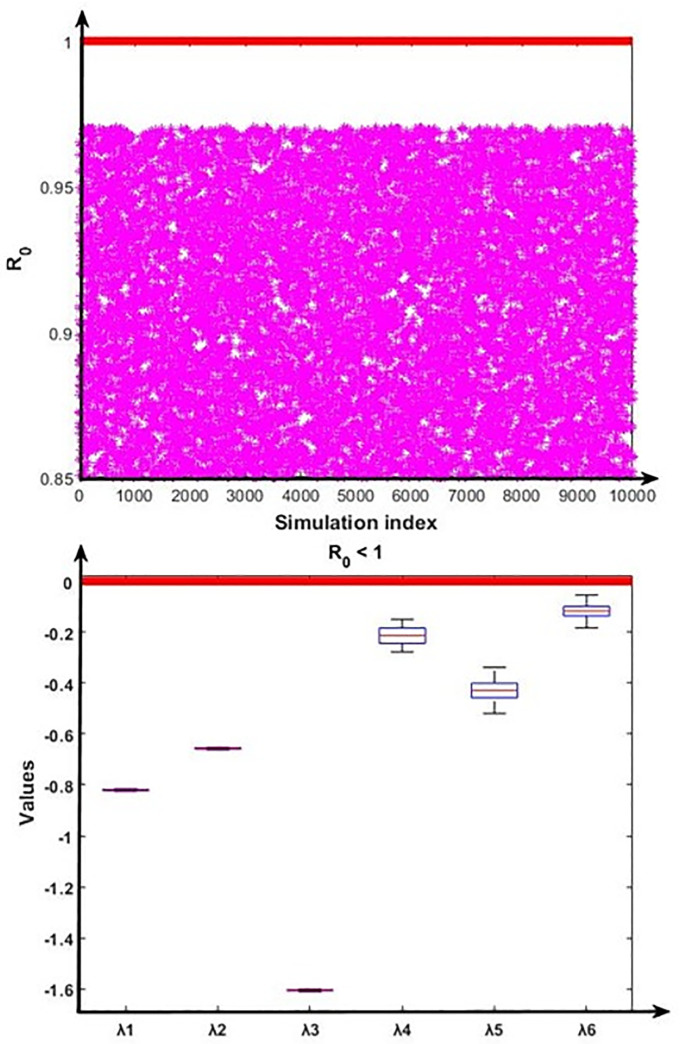
Numerical simulation for the stability condition of the disease-free equilibrium and the eigenvalues (λ_1_, λ_2_, λ_3_, λ_4_, λ_5_ and λ_6_) real part distribution. First portion represents that R_0_ < 1 always hold, and second portion depict the eigenvalues real part related distribution of the disease-free conditions.

**Fig 3:**
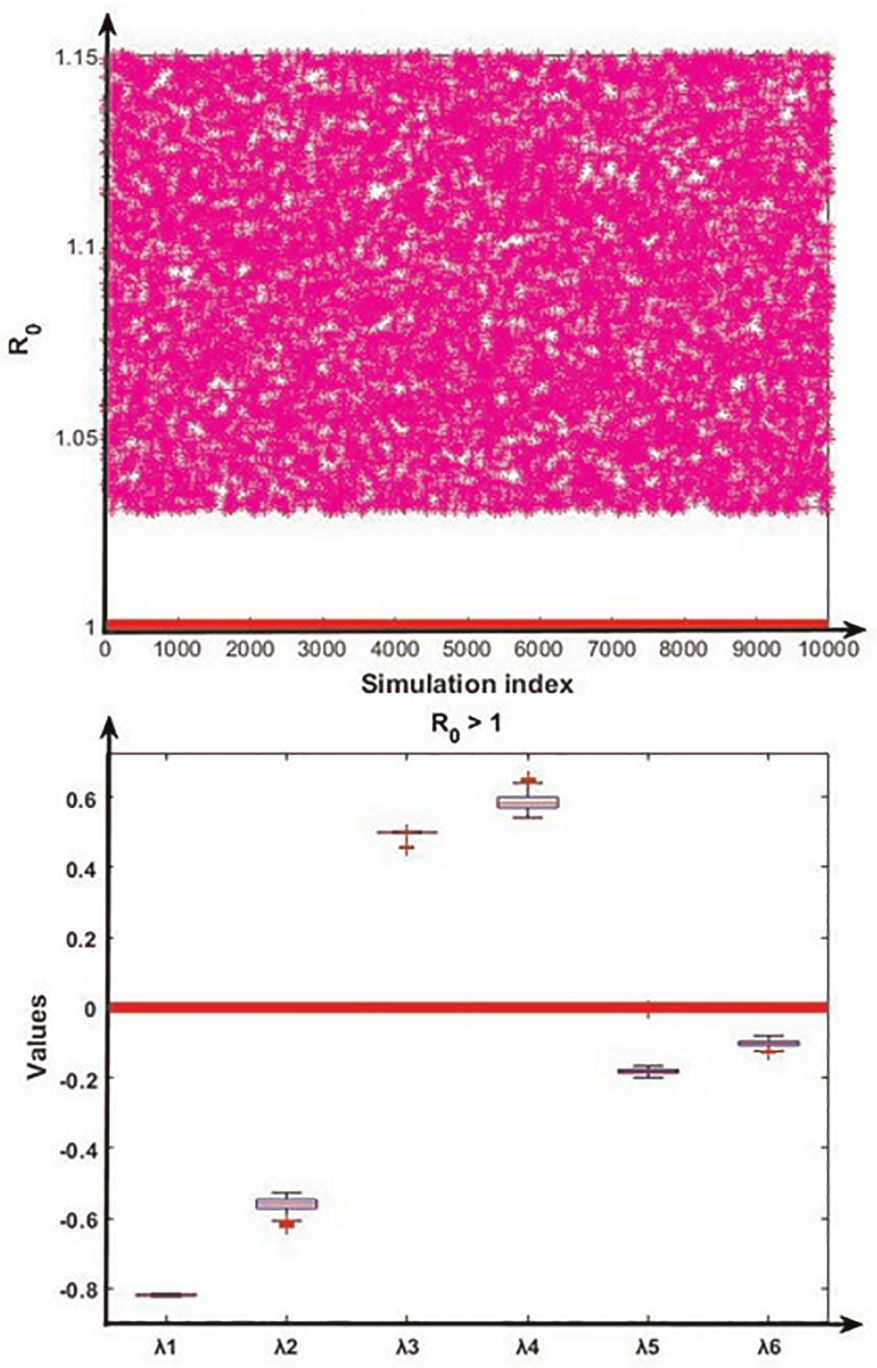
Numerical analysis for the stability condition of the endemic equilibrium and the eigenvalues (λ_1_, λ_2_, λ_3_, λ_4_, λ_5_ and λ_6_) real part distribution. First portion represents that R_0_ < 1 always hold, and second portion depict the eigenvalues’ real part related distribution of the endemic equilibrium conditions.

To validate the nature of the disease-free and endemic equilibrium analysis, we conducted the numerical analysis using the Monte Carlo simulation method [56] to verify the stability properties for the disease-free equilibrium and endemic equilibrium by calculating the fundamental part of the eigenvalues of the Jacobian matrix of the system (10)–(15). According to the disease-free equilibrium and disease endemic equilibrium, the steady states coordinates can be expressed in terms of fourteen model parameters (β, η, μ, ρ, σ, κ, α_1_, α_2_, ω_1_, ω_2_, ϕ, γ_1_, γ_2_, δ) whose baseline values are given in Table 1. The pool of sampling $N=\prod_{k=1}^{14}A_{k}\in R_{+}^{14}$ was introduced by a Cartesian product of fourteen closed intervals of the form *A*_*k*_ = [*a*_*k*_ − *θa*_*k*_, *a*_*k*_ + *θa*_*k*_], where each *a*_*k*_, *k* = 1, 2, ……14 stands for the baseline value of one parameter (see Table 1), and *θ* > 0 represents the range of variation. Our sampling comprised 10,000 confounding scenarios $N=(a_{1},\ldots \ldots a_{14})\in N$ where each *a*_*k*_ ∈ *A*_*k*_, *k* = 1,2, ……14 was randomly chosen for *θ* = 0.20 (i.e., 20% deviation from the parameters baseline values) under a uniform distribution with no correlation between model parameters. The outcomes of this simulation are presented in Figs 2 and 3.

Fig 2 displayed that the disease-free equilibrium is locally asymptotically stable as the real part of all six eigenvalues are negative (i.e. λ_1_ < 0, λ_2_ < 0, λ_3_ < 0, λ_4_ < 0, λ_5_ < 0 and λ_6_ < 0). Further, Fig 3 displayed that the endemic equilibrium is unstable as the real part of the two eigenvalues is positive (i.e. λ_3_ > 0 and λ_4_ > 0).

### Estimation of parameters

**Fig 4:**
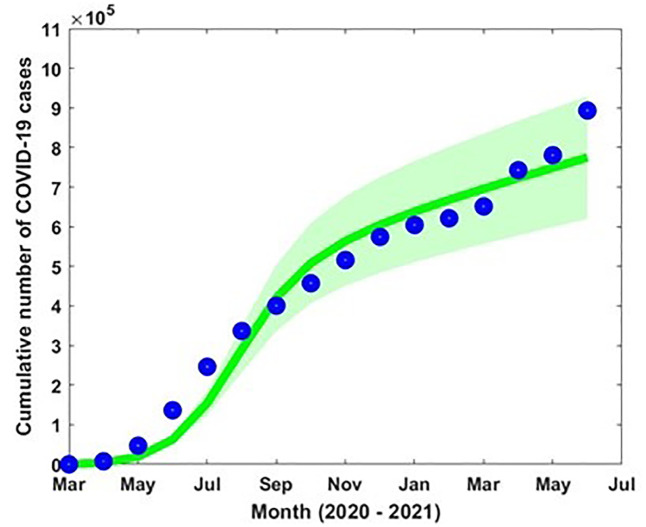
Reported COVID-19 incidence data (blue dots) and the model simulation (green solid curve), with the 95% confidence intervals (CI) indicated by the pink-shaded area.

Model parameters are estimated based on the accessible COVID-19 data from March 2020 to June 2021 in Bangladesh [57]. The incidence of COVID-19 data in Bangladesh was available, and the number of cases increased from May to July (2020–2021) due to the outbreak of different variants. The transmission rate β, progression rate from L to M and C (ω_1_ and ω_2_) and loss of immunities (*α*_1_ and *α*_2_) are estimated by minimizing the error of the incidence data to the model curve using the least-squares fitting technique which offers a superior fit [56]. We used MATLAB optimization routine (built-in function such as ‘fmincon’) to perform least-square fitting for parameter estimation. The ‘fmincon’ is a MATLAB function used for solving constrained optimization problems. It stands for "function minimization with constraints" and is part of MATLAB’s Optimization Toolbox. Initial guess is considered for the parameters to optimize. This is a starting point for the algorithm to begin the search. Fig 4 shows the incidence data (blue dot) and the model-fitted curve (green solid curve) with the estimated parameter values β = 2.34 × 10^−6^, ω_1_ = 0.013, ω_2_ = 0.00731, α_1_ = 0.0052, α_2_ = 0.0203 and η = 1.124.

In addition, the natural death rate (μ) was considered the inverse of the life expectancy (70 years) in Bangladesh. The rest of the parameter values were obtained from the literature (see Table 1).

The objective function used in the parameter estimation for the COVID-19 cumulative incidence is as follows:

$$\hat{\theta }=\text{argmin}\sum_{i=1}^{n}{\left(\left(\omega_{1}+\omega_{2}\right){\text{L}}_{{\text{t}}_{\text{i}}}-{\text{data}}_{\text{tip}}\right)}^{2}$$

where $dat{a_{t}}_{ip}$ represents the actual COVID-19 incidence data and $\left(\omega_{1}+\omega_{2}\right){L_{t}}_{i}$ is the corresponding model incidence solution at the time t_i_ and n is the number of available data points. The goal of the objective fuction is to minimize the sum of the squared differences (residuals) between the observed data and the model solution. The model (2)–(8) associated parameters are tabulated in Table 1.

### Sensitivity analysis

Sensitivity analysis is a crucial modeling technique that evaluates how changes in important parameters impact the model’s results. Sensitivity analysis offers a methodical way to manipulate one or more parameters and track changes in the output to determine which parameters influence the model’s behaviour most. Here, we performed the sensitivity analysis of R_0_ to the model parameters using the Latin Hypercube Sampling (LHS) method with 10000 runs per simulation. The LHS is a Monte Carlo stratified sampling method that allows us simultaneously to achieve an unbiased estimation of the model outcome for a specific set of input parameter values [51, 58, 59]. We allocated a uniform distribution for individual parameters from 0 to 4 times the baseline value.

**Fig 5:**
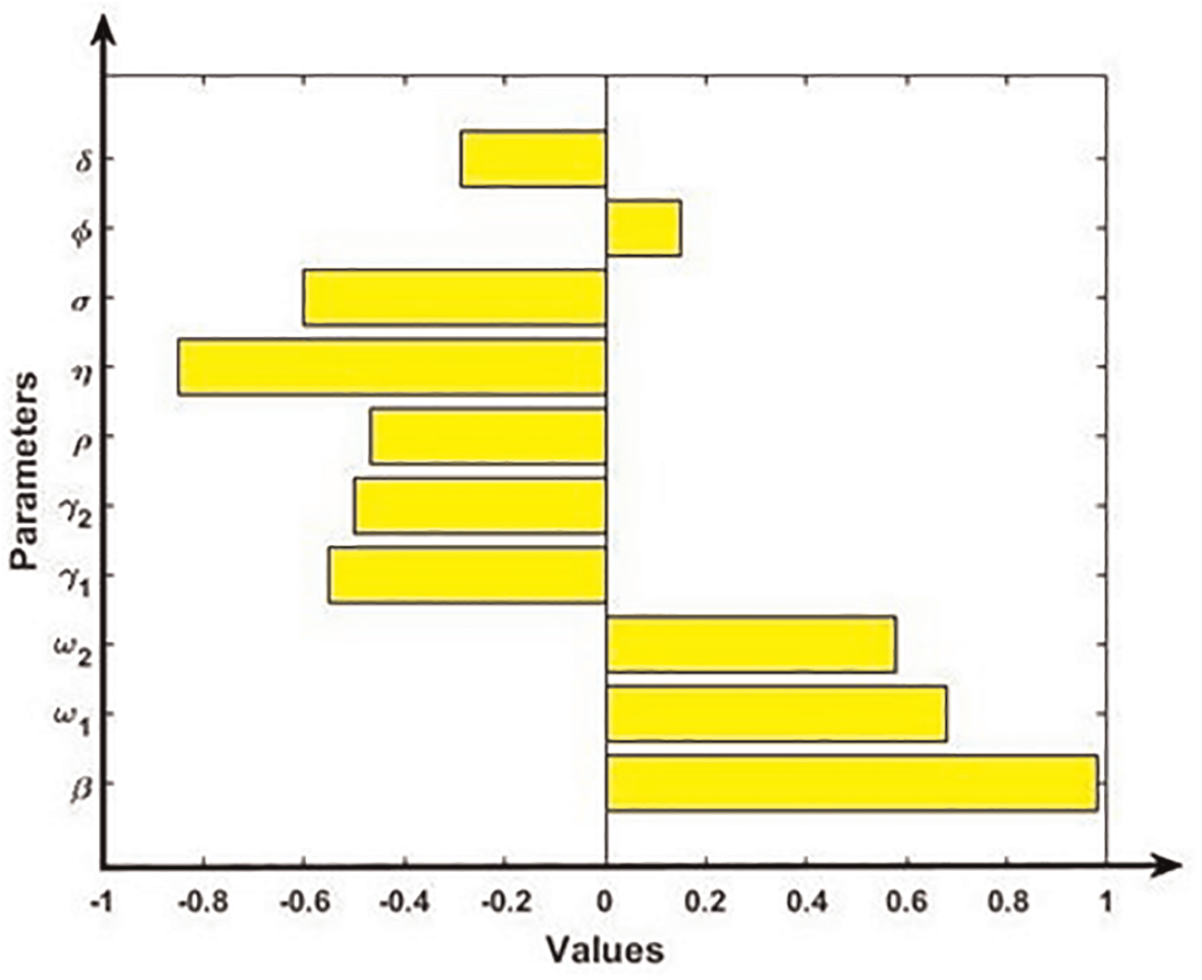
Sensitivity analysis of the model outcome R_0_ and model parameters β, ω_1_, ω_2_, γ_1_, γ_2_, ρ, η, σ, ϕ and δ.

We calculate the Partial Rank Correlation Coefficients (PRCCs), a global sensitivity analysis technique of the key output variables. The PRCC positive value of model parameters indicates a positive correlation with model outputs and negative values represent a negative correlation. The PRCC for the full range of parameters is shown in the tornado plots in Fig 5. Results show that parameters β, ω_1_, ω_2_ and ϕ have a positive correlation with the effective reproduction number R_0_, indicating that increasing the parameter values will increase the value of R_0_. We found that transmission rate (*β*) has the high positive impact on effective reproduction number R_0_. Hence, a small increase in the transmission rate can significantly raise the *R*_0_, leading to a more rapid spread of the disease and higher peak infection rates. Conversely, a reduction in transmission rate (*β*) through interventions can flatten the epidemic curve. On the contrary, parameters γ_1_, γ_2_, ρ, η, σ, and δ have a negative association with the effective reproduction number R_0_, implying that increasing the parameter values will reduce the value of R_0_. Here, we found that first dose vaccination rate (*η*) has the high negative impact on *R*_0_. Hence, the first does vaccination coverage rate significantly impacts herd immunity thresholds. Higher coverage rates lead to lower overall infection rates and can prevent outbreaks from reaching epidemic levels.

### Bifurcation analysis

In this section, we present the bifurcation analysis of the proposed model (2)–(8) using the centre manifold theory as delineated by Castillo-Chavez and Song [60] (Theorem 4.1). We assume the bifurcation parameter as β, R_0_ = 1, and then perform the bifurcation analysis [60–63]. We rewrite the model by considering S = x_1_, V_1_ = x_2_, V_2_ = x_3_, L = x_4_, M = x_5_, C = x_6_ as **x** = (x_1_, x_2_, x_3_, x_4_, x_5_, x_6_)^T^, so the structure can be expressed as $\frac{dx}{dt}=f(x)$, where **f** = (f_1_, f_2_, f_3_, f_4_, f_5_, f_6_)^T^.

***Theorem 3***

The model proposed in (2)–(8) has experienced backward bifurcation at β = β*, R_0_ = 1. We provided the relative expression in the proof.

***Proof:***

Let consider S = x_1_ and similarly, V_1_ = x_2_, V_2_ = x_3_, L = x_4_, M = x_5_, C = x_6_. Then the system can be expressed as:

$$\begin{array}{ll} \frac{{\text{dx}}_{1}}{\text{dt}}= & \mu \text{N}+{\rho \text{x}}_{2}+{\delta \text{x}}_{6}-{\beta \text{x}}_{1}\left({\text{x}}_{5}+{\text{x}}_{6}\right)-{\text{v}}_{1}{\text{x}}_{1}\\ \frac{{\text{dx}}_{2}}{\text{dt}}= & {\beta \text{x}}_{1}-{\text{v}}_{2}{\text{x}}_{2}\\ \frac{{\text{dx}}_{3}}{\text{dt}}= & {\sigma \text{x}}_{2}-{\text{v}}_{3}{\text{x}}_{3}\\ \frac{{\text{dx}}_{4}}{\text{dt}}= & {\beta \text{x}}_{1}\left({\text{x}}_{5}+{\text{x}}_{6}\right)+\alpha_{1}{\text{x}}_{2}+\alpha_{2}{\text{x}}_{3}-{\text{v}}_{4}{\text{x}}_{4}\\ \frac{{\text{dx}}_{5}}{\text{dt}}= & \omega_{1}{\text{x}}_{4}-{\text{v}}_{5}{\text{x}}_{5}\\ \frac{{\text{dx}}_{6}}{\text{dt}}= & \omega_{2}{\text{x}}_{4}+\phi {\text{x}}_{5}-{\text{v}}_{6}{\text{x}}_{6} \end{array}$$ (20)

Here, v_1_ = (η + μ), v_2_ = (ρ + σ + α_1_ + μ), v_3_ = (κ + α_2_ + μ), v_4_ = (ω_1_ + ω_2_ + μ), v_5_ = (γ_1_ + ϕ + μ), v_6_ = (γ_2_ + δ + μ), and v_7_ = u_1_u_2_–ηρ. The transmission rate among the susceptible, unvaccinated group is defined as the bifurcation parameter, with the condition R_0_ = 1. Thus, we have

$$\beta^{*}=\frac{\left(\left(\rho +\sigma ++\mu \right)\left(\eta +\mu \right)-\eta \rho \right)\left(\omega_{1}+\omega_{2}+\mu \right)\left(\phi +\gamma_{1}+\mu \right)\left(\gamma_{2}+\delta +\mu \right)}{\mu N\left(\rho +\sigma +\alpha_{1}+\mu \right)\left[\omega_{1}\left(\phi +\gamma_{2}+\delta +\mu \right)+\omega_{2}\left(\phi +\gamma_{1}+\mu \right)\right]}$$ (21)

So the disease-free equilibrium point for the transformed system (20) is,

$$E^{0}=\left(x_{1}^{0},x_{2}^{0},x_{3}^{0},x_{4}^{0},x_{5}^{0},x_{6}^{0}\right)=\left(\frac{v_{2}N\mu }{v_{7}},\frac{N\mu \eta }{v_{7}},\frac{N\mu \eta \sigma }{v_{7}},0,0,0\right)$$

The linearization matrix of the transformed system (20), can be expressed in terms of Jacobian as J(E^0^):

$$\text{J}\left({\text{E}}^{0}\right)=\left[\begin{array}{cccccc} -{\text{v}}_{1} & \rho & 0 & 0 & -\beta \frac{{\text{v}}_{2}\text{N}\mu }{{\text{v}}_{7}} & -\beta \frac{{\text{v}}_{2}\text{N}\mu }{{\text{v}}_{7}}+\delta \\ \eta & -{\text{v}}_{2} & 0 & 0 & 0 & 0\\ 0 & \sigma & -{\text{v}}_{3} & 0 & 0 & 0\\ 0 & \alpha_{1} & \alpha_{2} & -{\text{v}}_{4} & \beta \frac{{\text{v}}_{2}\text{N}\mu }{{\text{v}}_{7}} & \beta \frac{{\text{v}}_{2}\text{N}\mu }{{\text{v}}_{7}}\\ 0 & 0 & 0 & \omega_{1} & -{\text{v}}_{5} & 0\\ 0 & 0 & 0 & \omega_{2} & \phi & -{\text{v}}_{6} \end{array}\right]$$ (22)

When, R_0_ = 1, i.e., at β*, the expressed Jacobian (22) of the system possesses a single zero eigenvalue, and the remaining eigenvalues exhibit negative real parts. Consequently, the behavior of the transformed system in the vicinity of β* (at R_0_ = 1) is analyzed through the application of central manifold theory [60]. The essential computations based on the central manifold theory [60–63] are outlined as follows. Let. **H** = (h_1,_ h_2,_ h_3,_ h_4,_ h_5,_ h_6_)^T^ be the right eigenvector for J(E^0^) at R_0_ = 1, is determined by solving J(E^0^).**H** = 0, and therefore

h1=h2α1+ρv3+α2σμβv6+ϕω1+v5ω2Nv2-v7ϕδα2σ+α1v3ω1+δα1ω2+ρv4v6v3+α2σδω2v5v1v3μβv6+ϕω1+v5ω2Nv2-v4v5v6v7,

$$h_{3}=\frac{h_{2}\sigma }{v_{3}},h_{4}=\frac{h_{2}v_{5}v_{6}v_{7}\left(\alpha_{2}\sigma +{\alpha_{1}v}_{3}\right)}{v_{3}\left(-\mu \beta \left(\left(v_{6}+\phi \right)\omega_{1}+{v_{5}\omega }_{2}\right)Nv_{2}+v_{4}v_{5}v_{6}v_{7}\right)},$$

$$h_{5}=\frac{h_{2}\omega_{1}v_{6}v_{7}\left(\alpha_{2}\sigma +{\alpha_{1}v}_{3}\right)}{v_{3}\left(-\mu \beta \left(\left(v_{6}+\phi \right)\omega_{1}+{v_{5}\omega }_{2}\right)Nv_{2}+v_{4}v_{5}v_{6}v_{7}\right)},$$

$$h_{6}=\frac{h_{2}\left(\phi \omega_{1}+{v_{5}\omega }_{2}\right)v_{7}\left(\alpha_{2}\sigma +{\alpha_{1}v}_{3}\right)}{v_{3}\left(-\mu \beta \left(\left(v_{6}+\phi \right)\omega_{1}+{v_{5}\omega }_{2}\right)Nv_{2}+v_{4}v_{5}v_{6}v_{7}\right)}.$$

Let. **K** = (k_1,_ k_2,_ k_3,_ k_4,_ k_5,_ k_6_)^T^ be the left eigenvector for J(E^0^) at R_0_ = 1, is determined by solving **K**.J(E^0^) = 0 (calculated by J(E^0^)^T^. **K**), and therefore we have,

$$k_{1}=\frac{\eta k_{2}}{v_{1}},k_{3}=\frac{\eta k_{2}\alpha_{2}\left(\mu \left(\left(v_{6}+\phi \right)\omega_{1}+{v_{5}\omega }_{2}\right)\beta Nv_{2}-\delta v_{7}\left(\phi \omega_{1}+{v_{5}\omega }_{2}\right)\right)}{v_{1}v_{3}\left(\mu \beta \left(\left(v_{6}+\phi \right)\omega_{1}+{v_{5}\omega }_{2}\right)Nv_{2}-v_{4}v_{5}v_{6}v_{7}\right)},$$

$$k_{4}=\frac{\eta k_{2}\left(\mu \left(\left(v_{6}+\phi \right)\omega_{1}+{v_{5}\omega }_{2}\right)\beta Nv_{2}-\delta v_{7}\left(\phi \omega_{1}+{v_{5}\omega }_{2}\right)\right)}{v_{1}\left(\mu \beta \left(\left(v_{6}+\phi \right)\omega_{1}+{v_{5}\omega }_{2}\right)Nv_{2}-v_{4}v_{5}v_{6}v_{7}\right)},$$

$$k_{5}=\frac{\eta k_{2}\left(\mu \left(\left(v_{6}+\phi \right)v_{4}-{\delta \omega }_{2}\right)\beta Nv_{2}-\delta {\phi v_{4}v}_{7}\right)}{v_{1}v_{3}\left(\mu \beta \left(\left(v_{6}+\phi \right)\omega_{1}+{v_{5}\omega }_{2}\right)Nv_{2}-v_{4}v_{5}v_{6}v_{7}\right)},$$

$$k_{6}=\frac{\eta k_{2}\left(\beta Nv_{2}\mu \left(\delta \omega_{1}+v_{4}v_{5}\right)-\delta v_{4}v_{5}v_{7}\right)}{v_{1}\left(\mu \beta \left(\left(v_{6}+\phi \right)\omega_{1}+{v_{5}\omega }_{2}\right)Nv_{2}-v_{4}v_{5}v_{6}v_{7}\right)}.$$

Subsequently, k_2_ is computed to satisfy the requirement that **H**.**K** = 1^56^, thus resulting in k_2_ = 1, whereas h_2_ can be expressed as:

$${\text{h}}_{2}=\frac{1}{\left(\begin{array}{c} 1+\frac{\eta \left(\begin{array}{c} \left(\left(\alpha_{1}+\rho \right){\text{v}}_{3}+\alpha_{2}\sigma \right)\mu \beta \left(\left({\text{v}}_{6}+\phi \right)\omega_{1}+{\text{v}}_{5}\omega_{2}\right)N{\text{v}}_{2}\\ -{\text{v}}_{7}\left(\phi \delta \left(\alpha_{2}\sigma +\alpha_{1}{\text{v}}_{3}\right)\omega_{1}+\left(\left({\delta \alpha }_{1}\omega_{2}+{\rho \text{v}}_{4}{\text{v}}_{6}\right){\text{v}}_{3}+\alpha_{2}{\sigma \delta \omega }_{2}\right){\text{v}}_{5}\right) \end{array}\right)}{{\text{v}}_{1}{\text{v}}_{3}\left(\mu \beta \left(\left({\text{v}}_{6}+\phi \right)\omega_{1}+{\text{v}}_{5}\omega_{2}\right){\text{Nv}}_{2}-{\text{v}}_{4}{\text{v}}_{5}{\text{v}}_{6}{\text{v}}_{7}\right){\text{v}}_{1}}\\ +\frac{{\eta \alpha }_{2}\sigma \left(\mu \left(\left({\text{v}}_{6}+\phi \right)\omega_{1}+{\text{v}}_{5}\omega_{2}\right){\beta \text{Nv}}_{2}-{\delta \text{v}}_{7}\left(\phi \omega_{1}+{\text{v}}_{5}\omega_{2}\right)\right)}{{\text{v}}_{1}{\text{v}}_{3}\left(\mu \beta \left(\left({\text{v}}_{6}+\phi \right)\omega_{1}+{\text{v}}_{5}\omega_{2}\right){\text{Nv}}_{2}-{\text{v}}_{4}{\text{v}}_{5}{\text{v}}_{6}{\text{v}}_{7}\right){\text{v}}_{3}}\\ -\frac{\eta \left({\text{v}}_{5}{\text{v}}_{6}{\text{v}}_{7}\left(\alpha_{2}\sigma +\alpha_{1}{\text{v}}_{3}\right)\right)\left(\mu \left(\left({\text{v}}_{6}+\phi \right)\omega_{1}+{\text{v}}_{5}\omega_{2}\right){\beta \text{Nv}}_{2}-{\delta \text{v}}_{7}\left(\phi \omega_{1}+{\text{v}}_{5}\omega_{2}\right)\right)}{{\text{v}}_{1}{\text{v}}_{3}{\left(\mu \beta \left(\left({\text{v}}_{6}+\phi \right)\omega_{1}+{\text{v}}_{5}\omega_{2}\right){\text{Nv}}_{2}-{\text{v}}_{4}{\text{v}}_{5}{\text{v}}_{6}{\text{v}}_{7}\right)}^{2}}\\ -\frac{\eta \left(\omega_{1}{\text{v}}_{6}{\text{v}}_{7}\left(\alpha_{2}\sigma +\alpha_{1}{\text{v}}_{3}\right)\right)\left(\mu \left(\left({\text{v}}_{6}+\phi \right){\text{v}}_{4}-{\delta \omega }_{2}\right){\beta \text{Nv}}_{2}-\delta \phi {\text{v}}_{4}{\text{v}}_{7}\right)}{{\text{v}}_{3}{\text{v}}_{1}{\text{v}}_{3}{\left(\mu \beta \left(\left({\text{v}}_{6}+\phi \right)\omega_{1}+{\text{v}}_{5}\omega_{2}\right){\text{Nv}}_{2}-{\text{v}}_{4}{\text{v}}_{5}{\text{v}}_{6}{\text{v}}_{7}\right)}^{2}}\\ -\frac{{\eta \text{v}}_{7}\left({\beta \text{Nv}}_{2}\mu \left({\delta \omega }_{1}+{\text{v}}_{4}{\text{v}}_{5}\right)-{\delta \text{v}}_{4}{\text{v}}_{5}{\text{v}}_{7}\right)\left(\phi \omega_{1}+{\text{v}}_{5}\omega_{2}\right)\left(\alpha_{2}\sigma +\alpha_{1}{\text{v}}_{3}\right)}{{\text{v}}_{1}{\text{v}}_{3}{\left(\mu \beta \left(\left({\text{v}}_{6}+\phi \right)\omega_{1}+{\text{v}}_{5}\omega_{2}\right){\text{Nv}}_{2}-{\text{v}}_{4}{\text{v}}_{5}{\text{v}}_{6}{\text{v}}_{7}\right)}^{2}} \end{array}\right)},$$

So, the bifurcation coefficient **a** and **b** can be written as^55,56,57,58^,

$$a=\overset{6}{\underset{\text{p},\text{q},\text{r}=1}{\sum }}{\text{k}}_{\text{r}}{\text{h}}_{\text{p}}{\text{h}}_{\text{q}}\frac{\partial^{2}{\text{f}}_{\text{r}}\left({\text{E}}^{0},\beta^{*}\right)}{\partial {\text{x}}_{\text{p}}\partial {\text{x}}_{\text{q}}}\;\text{and}\;b=\overset{6}{\underset{\text{p},\text{q},\text{r}=1}{\sum }}{\text{k}}_{\text{r}}{\text{h}}_{\text{p}}\frac{\partial^{2}{\text{f}}_{\text{r}}\left({\text{E}}^{0},\beta^{*}\right)}{\partial {\text{x}}_{\text{p}}\partial \beta }.$$ (23)

**Fig 6:**
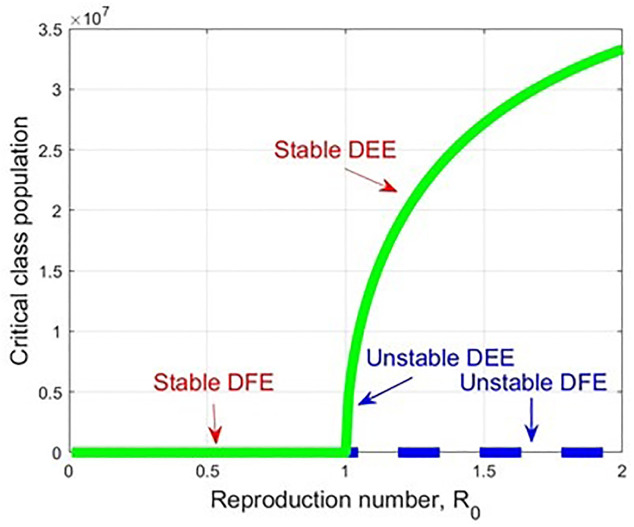
A forward bifurcation graph which displays the stability changes concerning R_0_, when R_0_ ranges from 0 to 2.

By substituting the corresponding values of H = (h_1,_ h_2,_ h_3,_ h_4,_ h_5,_ h_6_), K = (k_1,_ k_2,_ k_3,_ k_4,_ k_5,_ k_6_), and performing the partial derivative of the transformed function (f_l_(E^0^, β*)), putting the corresponding rate parameter values, we observe the forward bifurcation at R_0_ = 1. In the forward bifurcation case, the disease-free equilibrium becomes unstable, and the disease-endemic equilibrium point becomes stable when R_0_ >1 (see Fig 6). Forward bifurcation provides a clear and predictable threshold behaviour at *R*_0_ = 1, making it easy to determine whether COVID-19 will die out or become endemic. It indicates that maintaining R_0_ below 1 is sufficient to eliminate the disease. This insight helps in designing control strategies that are focused on reducing *R*_0_ through vaccination, social distancing, or other interventions.

### Numerical simulation

In this section, we carry out detailed numerical simulations using the MATLAB programming language to support the analytic results and measure the effect of first and second-dose vaccination and different biological parameters on the proposed COVID-19 model in Bangladesh. MATLAB’s powerful numerical solvers and visualization tools make it ideal for analyzing the behavior of epidemic models across various domains. We used the MATLAB toolbox to solve ordinary differential equations (ODEs). It has built-in functions like ‘ode45’ for solving ODEs of our proposed model. For illustration, we have chosen baseline parameter values consistent with COVID-19 infection and transmission. By the analytic results, we found two equilibrium points: the infection-free equilibrium (E^0^); and disease endemic equilibrium(E*). Furthermore, we employed various initial conditions for all populations and established that if R_0_ is less than one, then the infection-free equilibrium is locally asymptotically stable. Further, if R_0_ is greater than one, then the COVID-19 disease persists in the population.

**Fig 7:**
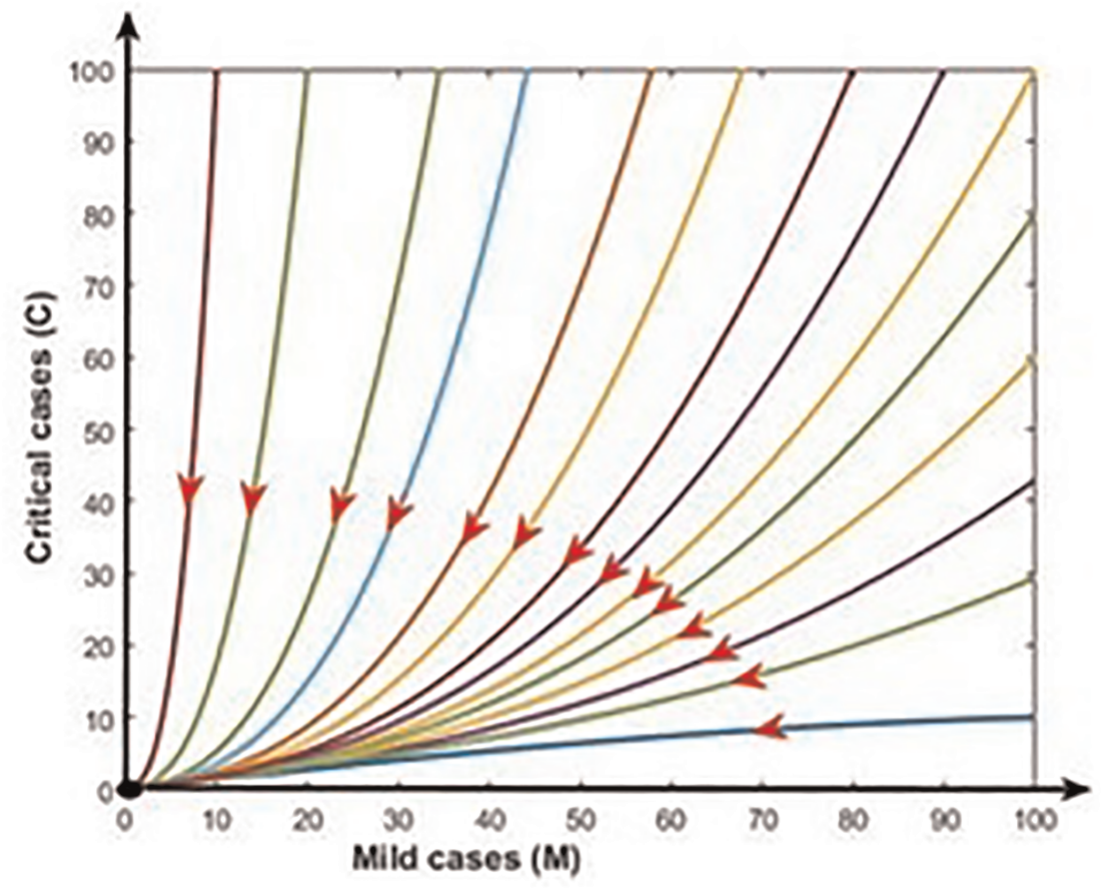
Disease free equilibrium: R_0_ < 1. In this case COVID-19 fade-out (black dot).

**Fig 8:**
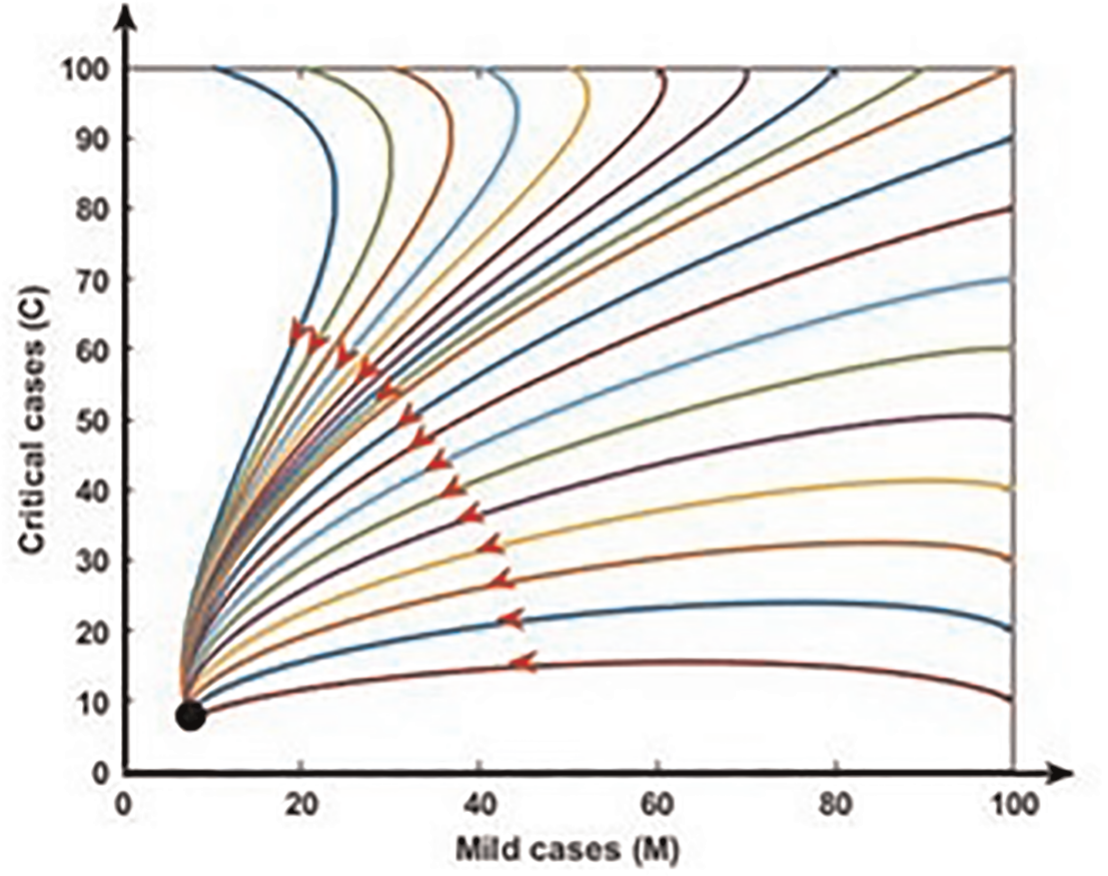
Endemic equilibrium: R_0_ > 1. In this case COVID-19 persist in the population (black dot).

Fig 7 demonstrates the stability of the infection-free equilibrium (i.e., R_0_ < 1) by illustrating model trajectories through the M vs C plane starting for various initial conditions. In such a case, COVID-19 fade-out. Fig 8 shows the stability of the endemic equilibrium (i.e., R_0_ > 1), and in that event, COVID-19 disease persists in the population.

**Fig 9:**
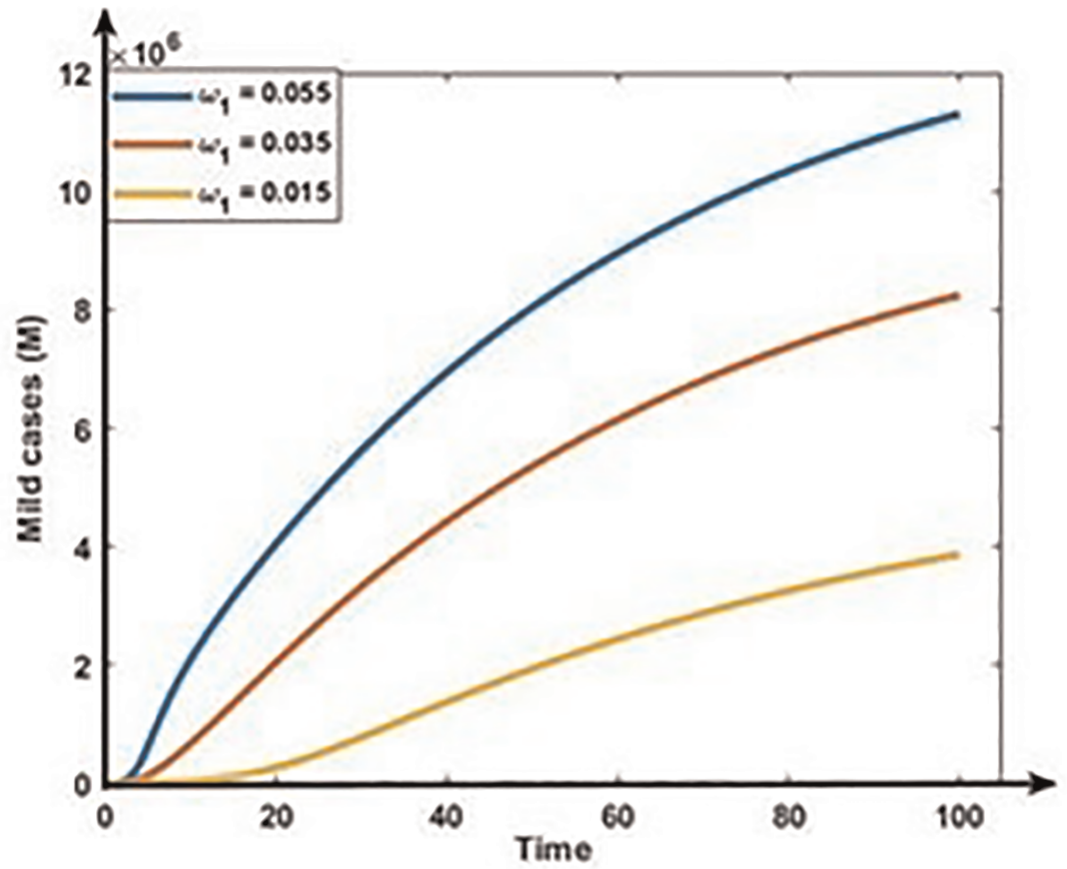
Impact of progression rate (ω_1_) on the dynamics of Mild cases (M).

**Fig 10:**
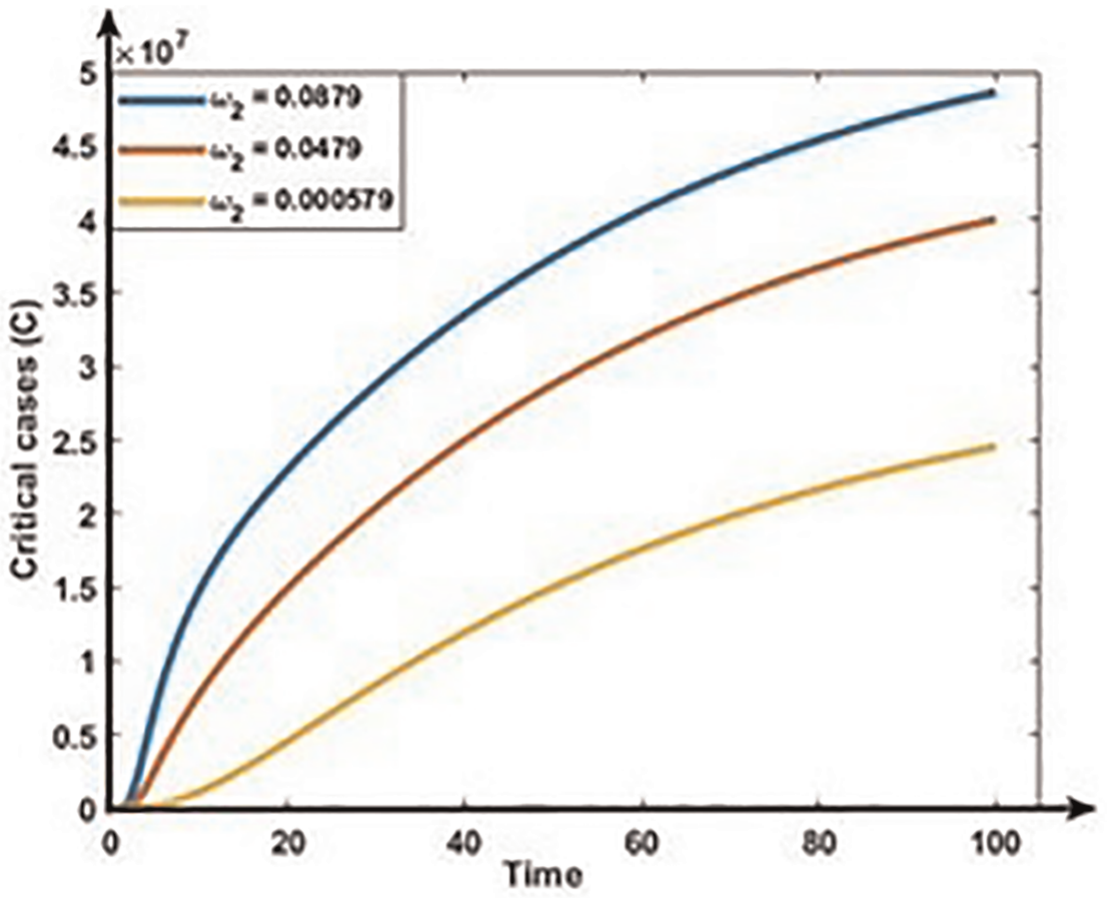
Impact of progression rate (ω_2_)on the dynamics of Critical cases (C).

Figs 9 and 10 show the impact of progression rates (ω_1_ and ω_2_) on the dynamics of Mild and Critical cases. From these Figures, we perceive that the burden of Mild and Critical cases increases if the progression rates rise, which means those positively associated with Mild and Critical cases.

**Fig 11:**
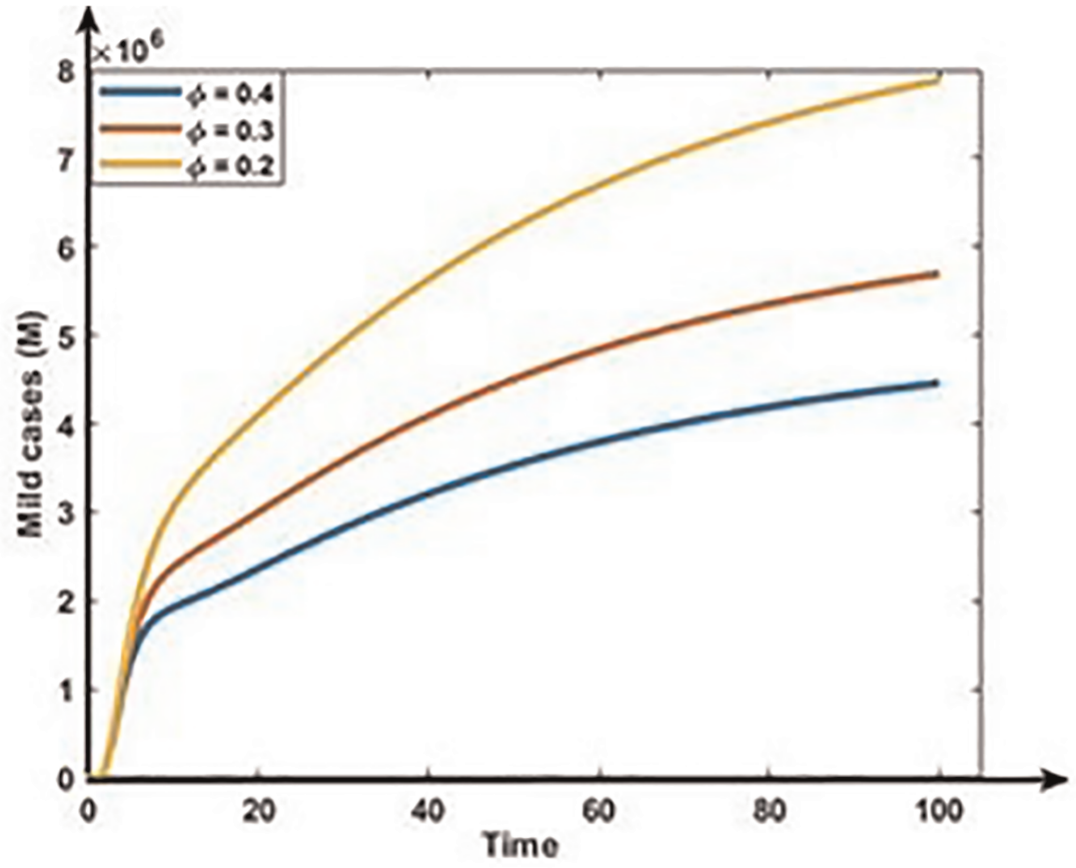
Impact of co-infection rate (ϕ) on the dynamics of Mild cases (M).

**Fig 12:**
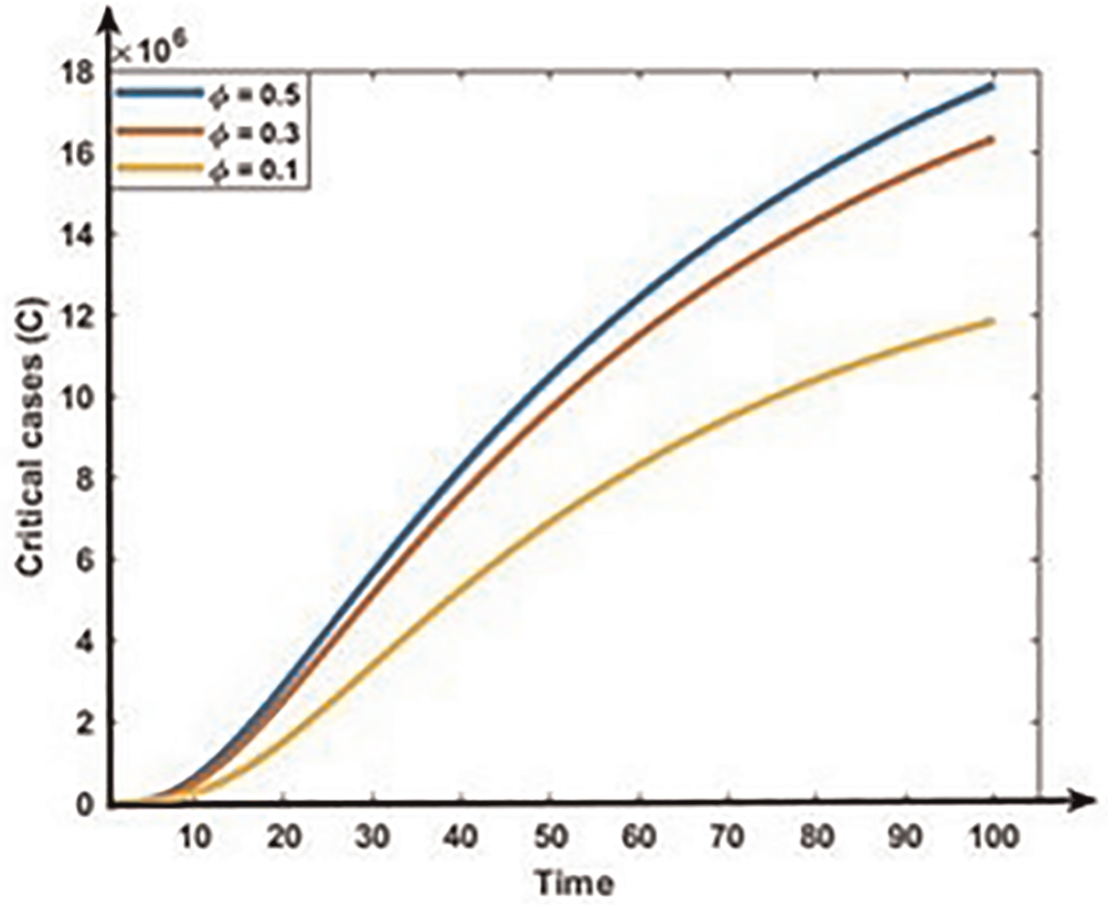
Impact of co-infection rate (ϕ) on the dynamics of Critical cases (C).

Figs 11 and 12 show the impact of co-infection rate on the dynamics of Mild and Critical cases. Results show that the co-infection rate negatively correlates with Mild cases but positively correlates with Critical cases. Our analysis is consistent with reality because the following infection, some Mild populations move to the critical cases due to the co-infection with other diseases, including hypertension, diabetes, cardiovascular disease, and respiratory disease.

**Fig 13:**
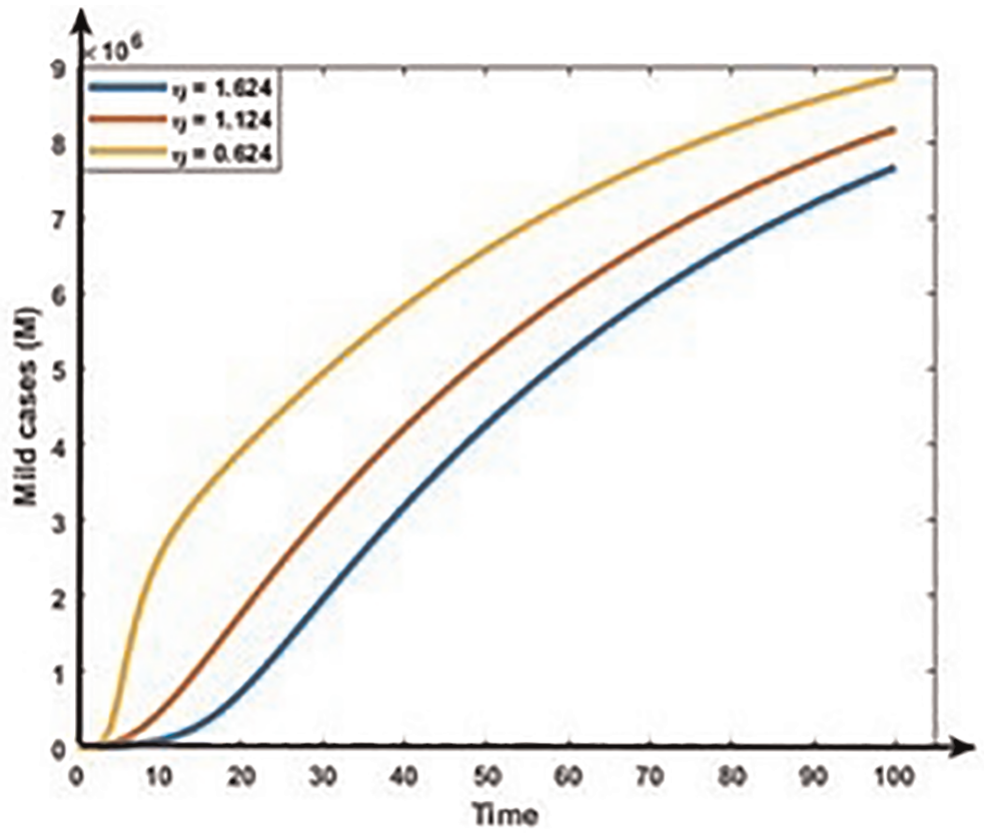
Impact of first does vaccination rate (η) on the dynamics of Mild cases (M).

**Fig 14:**
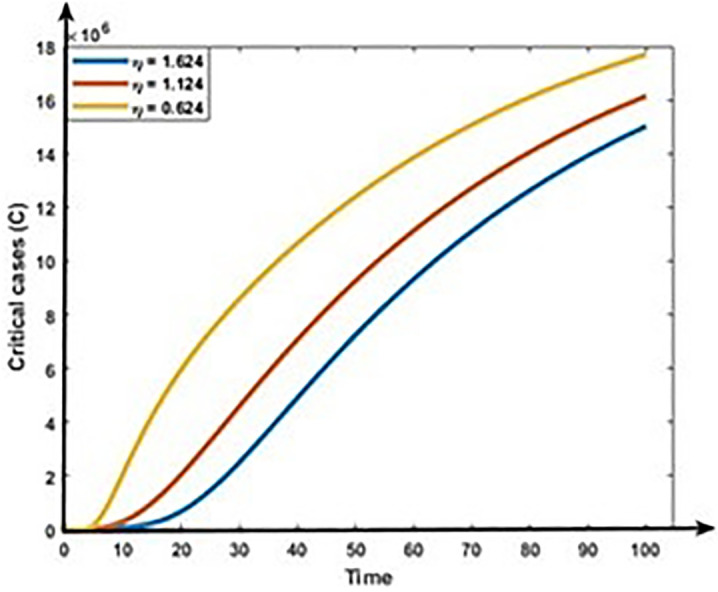
Impact of first does vaccination rate (η) on the dynamics of Critical cases (C).

**Fig 15:**
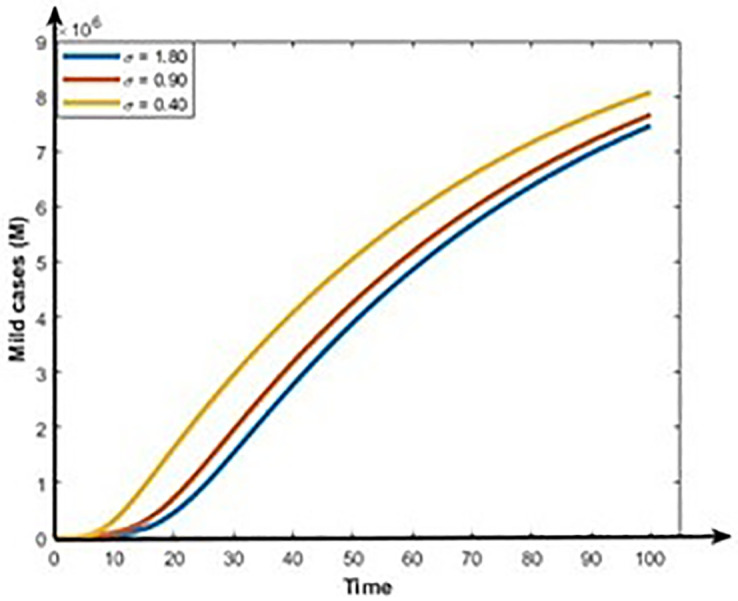
Impact of second does vaccination rate (σ) on the dynamics of Mild cases (M).

**Fig 16:**
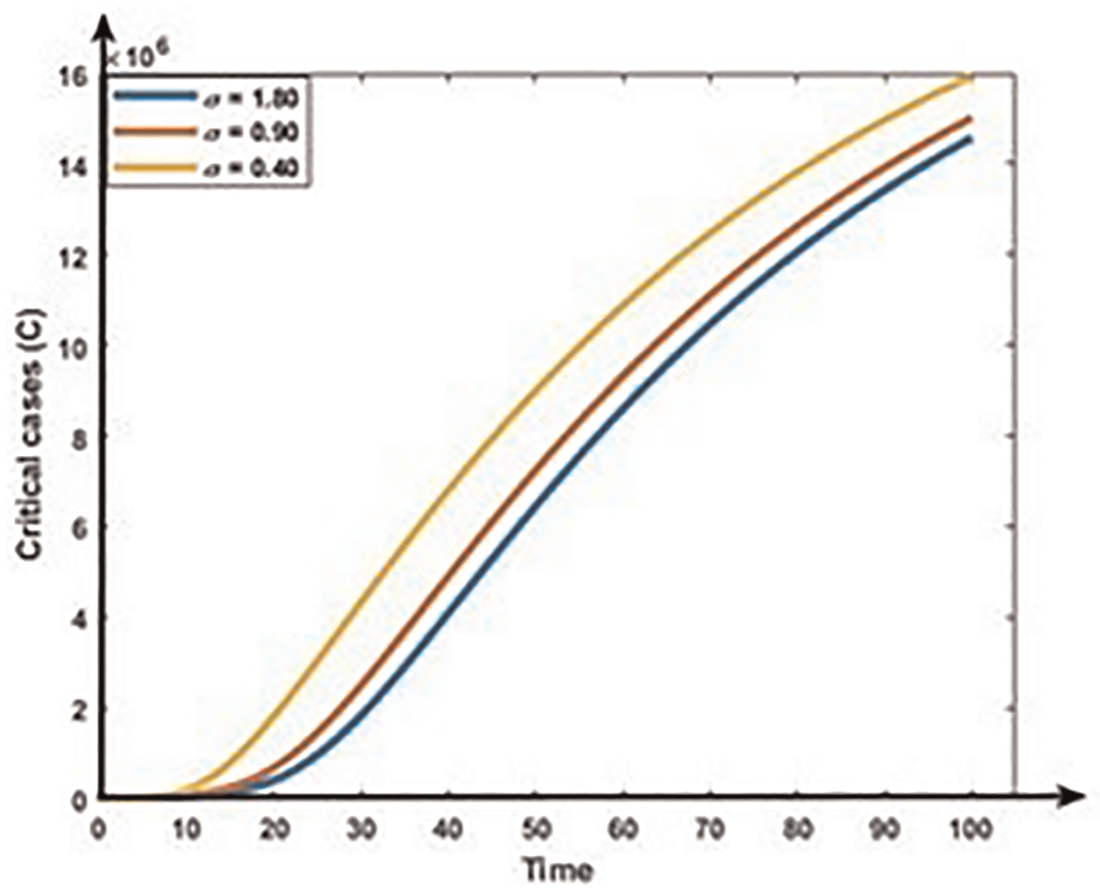
Impact of second does vaccination rate (σ) on the dynamics of Critical cases (C).

Figs 13 and 14 show that the rises in the first dose vaccination rates decrease the Mild and Critical cases of COVID-19 and decrease the risk of an outbreak. Figs 15 and 16 display that the rise in the second dose vaccination rates declines the Mild and Critical cases of COVID-19 in Bangladesh. From this analysis, we observe that the First-dose vaccination rate has a higher impact on COVID-19 cases compared to the second-dose vaccination rate, which is consistent with our observation and previous study [64] because the first-dose vaccine may prevent transmission from an infected person to another person.

## Discussion and conclusion

In this paper, we proposed a seven-compartmental COVID-19 model by introducing the epidemiology of mild and critical cases with dual-dose vaccination in Bangladesh. We analyzed that the solutions of this model are bounded and non-negative. We also determined a mathematical expression for the effective reproduction number of the model to confirm whether this disease persists or dies out. We found that if R_0_ is less than one, i.e., R_0_ < 1, the disease-free equilibrium is locally asymptotically stable. Conversely, if R_0_ is greater than one, i.e., R_0_ > 1, the coronavirus infection persists in the population. This analysis can help us to identify regions in the parameter space where the various asymptotic states are stable or unstable, thus allowing us to predict the long-term behavior of the COVID-19 dynamics. This information can advise the Ministry of Health in Bangladesh to reduce the period of infectiousness until *R*_0_ < 1.

The existence and stability of the transmission dynamics of nonlinear COVID-19 models were considered in previous modeling studies [45, 46, 65–68]. Previous studies show that if the effective reproduction number is less than one, the COVID-19 disease dies out of the community. On the other hand, if the effective reproduction number is greater than one, the disease persists in the community [45, 46, 66, 67], which is similar to our results. Numerous COVID-19 modelling studies examined the effect of different intervention scenarios. Results show that combined intervention strategies are the most effective for reducing the burden of COVID-19. In this study, we considered Bangladesh-specific seven compartmental COVID-19 model and allowed the Mild class population to move to the Critical class due to the co-infection and comorbidity with other diseases, including diabetes, cardiovascular disease, chronic respiratory disease, and cancer are more likely to develop a serious illness which is not considered in previous modeling studies [46, 48].

To investigate the significance of the model parameters, we implemented the sensitivity analysis of the effective reproduction number R_0_ corresponding to the model parameters. The result shows that transmission rate β is the most significant parameter, which is because infectious COVID-19 patients transmit the disease through coughing, sneezing, and spitting are the main source of infection, which is consistent with previous modeling studies [36, 49, 58, 65]. If the inhaled coronavirus enters and settles in the body of a healthy person and begins to multiple infection. Therefore, it is crucial to defend susceptible persons from COVID-19 infected person from a public health viewpoint by efficiently dropping the transmission rate between susceptible and infectious persons. In addition, the degree of exposure to this disease is extensive for those who are in close and prolonged contact with an infectious individual. Hence, it is very important to identify and notify individuals who have been in contact with infected persons to break the chain of transmission. Controlling the transmission of COVID-19 involves implementing a combination of public health measures and individual behaviors to reduce the spread of the virus. The second most important parameter is the first dose vaccination rate η, which is because it prevents the spread of contagious and dangerous. WHO stated that vaccination not only protects vaccinated persons, it also reduces exposure in the community. Therefore, increasing completion of vaccination rate is the most effective way of preventing COVID-19 disease in Bangladesh.

Qualitative techniques are important to understand real-world phenomena, which offer valuable insights into public hesitancy, misinformation, cultural beliefs, and barriers to vaccination that quantitative methods often overlook. Understanding these factors is essential for developing effective communication strategies and interventions to enhance vaccine uptake in Bangladesh. Meanwhile, bifurcation analysis identifies critical points where minor changes in vaccination rates or public health measures can cause major shifts in disease dynamics, such as transitioning from a controlled outbreak to an epidemic or vice versa. This information is crucial for determining when to adjust vaccination strategies or modify public health measures.

Integrating qualitative and bifurcation analysis enables a comprehensive understanding of both social dynamics and the mathematical modeling of disease transmission. This interdisciplinary approach combines insights from social sciences and epidemiological modeling, improving the reliability of results by considering multiple dimensions of the problem. Justifying the use of both methodologies bridges the gap between theoretical modeling and practical realities, which is key for translating insights into actionable public health strategies in Bangladesh.

Determining the effective reproduction number can help public health officials focus vaccination efforts on areas with higher transmission rates. Our study’s insights into factors affecting transmission, such as co-infection, vaccination, and progression rates, can inform the design of public health interventions tailored to specific local contexts. Additionally, findings from bifurcation and stability analyses can guide policymakers on when to adjust control measures like lockdowns, social distancing, or mask mandates based on real-time data, thereby minimizing COVID-19 spread and reducing the socio-economic impact of extended restrictions. The study identifies key parameters, such as vaccination and transmission rates, significantly affecting disease dynamics. Public health authorities can use this information to allocate vaccines more effectively, prioritizing high-risk groups or areas with high transmission. This study also guides policymakers in adjusting strategies like testing, contact tracing, quarantine, or social distancing by pinpointing critical points where small changes in vaccination or health measures impact transmission. Additionally, the findings support contingency planning by anticipating scenarios where new variants with different transmission rates could arise.

From the numerical analysis, it is also clear that an increment in the vaccine dose rate reduces the spread of COVID-19, which is similar to the previous study [64]. Therefore, to control and eradicate COVID-19 infection, it is vital to consider the following policies: (a) the first and most significant policy is to reduce the transmission rate with infected people; (b) the second most significant policy is to raise the vaccination rate. Therefore, we recommended that the most feasible and optimal strategy to eliminate COVID-19 in Bangladesh is to decrease the transmission rate through a mass vaccination campaign to cover most of the total population vaccination as soon as possible. There are many ways we can minimize the transmission rate, such as personal respiratory protection; this includes wearing masks by patients to reduce the dispersal of coronavirus when they talk, cough, or sneeze. Moreover, patients must be taught basic infection control measures, such as covering the nose and mouth during coughing.

Ongoing research is crucial for developing and improving new vaccines, especially as the virus evolves with new variants. This includes studying long-term immunity, booster needs, and vaccine efficacy against emerging strains. To lessen the severity of COVID-19 infections and avoid fatalities, research into antiviral medications, monoclonal antibodies, and other therapies is still essential. This involves exploring alternative applications for already-approved medications and creating novel treatments based on the most recent research. International collaboration ensures equitable vaccine access, especially in low- and middle-income countries, including Bangladesh. Sharing epidemiological data and research findings across countries and institutions accelerates the global response to COVID-19. Collaborative research projects across borders have led to rapid advancements in understanding COVID-19, from vaccine development to public health strategies.

To balance the need to manage the virus and limit economic and social disturbance, decisions on public health measures, such as lockdowns, mask requirements, and travel restrictions, must be supported by scientific data. Governments may preserve public health and consider broader social effects by using evidence-based recommendations to guide their decision-making. In addition, stakeholders, policymakers, and researchers may all work together to improve the efficacy of the worldwide response to COVID-19 by utilizing study findings. Continued collaboration, informed decision-making, and translating research into practice are crucial to overcoming the challenges of the pandemic and preparing for future public health emergencies.

## Limitations

It is important to note that this study has several limitations. First, a homogeneous mixed population model was taken into consideration. Homogeneous models ignore variances in factors like age, behavior, health, and economic status, presuming uniformity throughout the population. These differences can substantially impact the dynamics of disease transmission, the results of treatments, and the efficacy of interventions. By including heterogeneous features into modeling techniques, public health policies may better accommodate the different requirements of the community while also improving forecast precision. Under-reporting and misclassification cases of COVID-19 occur in Bangladesh due to inadequate recognition of infectious disease surveillance, nonspecific COVID-19 symptoms, and restricted access to healthcare facilities, and bias cannot be completely eliminated. Therefore, more accurate data should be gathered in order to solve COVID-19-related difficulties. Before putting our findings into practice, policymakers must take data into consideration, as our suggested results are dependent on it. In future, we will develop a heterogeneous population model to explore how factors like age, health status, occupation and behaviour interact with COVID-19. We will also identify which subgroups are most at risk and design vaccination and targeted interventions.

## Supporting information

S1 Data (XLSX)
